# Socioeconomic risk markers of leprosy in high-burden countries: A systematic review and meta-analysis

**DOI:** 10.1371/journal.pntd.0006622

**Published:** 2018-07-09

**Authors:** Julia Moreira Pescarini, Agostino Strina, Joilda Silva Nery, Lacita Menezes Skalinski, Kaio Vinicius Freitas de Andrade, Maria Lucia F. Penna, Elizabeth B. Brickley, Laura C. Rodrigues, Mauricio Lima Barreto, Gerson Oliveira Penna

**Affiliations:** 1 Centro de Integração de Dados e Conhecimentos para Saúde (Cidacs), Fundação Oswaldo Cruz, Salvador, Brazil; 2 Department of Infectious Disease Epidemiology, London School of Hygiene & Tropical Medicine, London, United Kingdom; 3 Universidade Federal do Vale do São Francisco (UNIVASF), Paulo Afonso, Brazil; 4 Instituto de Saúde Coletiva, Universidade Federal da Bahia, Salvador, Brazil; 5 Universidade Estadual de Santa Cruz (UESC), Ilheus, Brazil; 6 Universidade Estadual de Feira de Santana (UEFS), Feira de Santana, Brazil; 7 Universidade Federal Fluminense, Instituto de Saúde da Comunidade, Niterói, Brazil; 8 Centro de Medicina Tropical, Universidade de Brasilia (UNB), Brasilia, Brazil; Swiss Tropical and Public Health Institute, SWITZERLAND

## Abstract

Over 200,000 new cases of leprosy are detected each year, of which approximately 7% are associated with grade-2 disabilities (G2Ds). For achieving leprosy elimination, one of the main challenges will be targeting higher risk groups within endemic communities. Nevertheless, the socioeconomic risk markers of leprosy remain poorly understood. To address this gap we systematically reviewed MEDLINE/PubMed, Embase, LILACS and Web of Science for original articles investigating the social determinants of leprosy in countries with > 1000 cases/year in at least five years between 2006 and 2016. Cohort, case-control, cross-sectional, and ecological studies were eligible for inclusion; qualitative studies, case reports, and reviews were excluded. Out of 1,534 non-duplicate records, 96 full-text articles were reviewed, and 39 met inclusion criteria. 17 were included in random-effects meta-analyses for sex, occupation, food shortage, household contact, crowding, and lack of clean (i.e., treated) water. The majority of studies were conducted in Brazil, India, or Bangladesh while none were undertaken in low-income countries. Descriptive synthesis indicated that increased age, poor sanitary and socioeconomic conditions, lower level of education, and food-insecurity are risk markers for leprosy. Additionally, in pooled estimates, leprosy was associated with being male (RR = 1.33, 95% CI = 1.06–1.67), performing manual labor (RR = 2.15, 95% CI = 0.97–4.74), suffering from food shortage in the past (RR = 1.39, 95% CI = 1.05–1.85), being a household contact of a leprosy patient (RR = 3.40, 95% CI = 2.24–5.18), and living in a crowded household (≥5 per household) (RR = 1.38, 95% CI = 1.14–1.67). Lack of clean water did not appear to be a risk marker of leprosy (RR = 0.94, 95% CI = 0.65–1.35). Additionally, ecological studies provided evidence that lower inequality, better human development, increased healthcare coverage, and cash transfer programs are linked with lower leprosy risks. These findings point to a consistent relationship between leprosy and unfavorable economic circumstances and, thereby, underscore the pressing need of leprosy control policies to target socially vulnerable groups in high-burden countries.

## Author summary

Many cases of leprosy still occur in low and middle-income countries, with a considerable proportion of them leading to permanent nerve damage and visible physical deformities. Disease elimination can be achieved with a better understanding of the sociodemographic characteristics of those most affected by the disease and by targeting those with greater risk within endemic countries. To address this question, we reviewed all published studies evaluating the social determinants of leprosy in countries endemic for leprosy. We found 39 studies, most of them conducted in Brazil (i.e., an upper-middle-income country), India or Bangladesh (i.e., lower-middle income countries), and none in low-income countries. Our review found strong evidence that males, household contacts of leprosy patients, individuals living in crowded households, and individuals who suffered food shortage in the past are more affected by leprosy. Evidence also exists that increasing age, poor sanitary and socioeconomic conditions, lower levels of education, and food insecurity are associated with a greater risk of leprosy. Our review underscores the importance of improving living conditions and decreasing inequality in low and middle-income countries to achieve leprosy elimination.

## Introduction

Leprosy, a chronic infectious disease caused by *Mycobacterium leprae*, remains endemic in 13 low and middle-income countries worldwide [1]. While effective and affordable multidrug therapies have the potential to cure infections, failures in detection and treatment can lead to the development of stigmatizing leprosy-associated grade-2 disabilities (G2Ds) [1, 2]. By recent estimates, 7% of the more than 200,000 new cases of leprosy detected each year occur in individuals who have already developed G2Ds by the time of diagnosis. To reduce the incidence of infection and prevent the onset of new G2Ds, the World Health Organization has advocated for targeted detection and intervention among higher risk groups within endemic countries [1, 3]. However, defining and intervening with the target groups at a subnational level remains a challenge due to a lack of understanding regarding the epidemiological risk markers of leprosy.

In recent years, there has been an increased recognition of the social determinants of health and of the potential of social interventions to enhance disease treatment and control strategies [4]. In the case of leprosy, existing evidence suggests that poor living conditions may be associated with increased risk, while the discrimination and fears associated with leprosy may lead to treatment delays, G2Ds, and decreases in individual economic productivity, thereby perpetuating poverty [5]. Recognizing this bidirectional association, several countries have made efforts to break the link between poverty and leprosy by incorporating poverty reduction efforts as a major component in health policies promoting leprosy control [6]. To better inform these health policies and to address residual gaps in knowledge related to the markers of leprosy risk, this systematic review aims to collate and appraise the published evidence on the effect of social, demographic, and economic factors and leprosy occurrence in high-burden settings.

## Methods

### Search strategy and eligibility criteria

**Fig 1:**
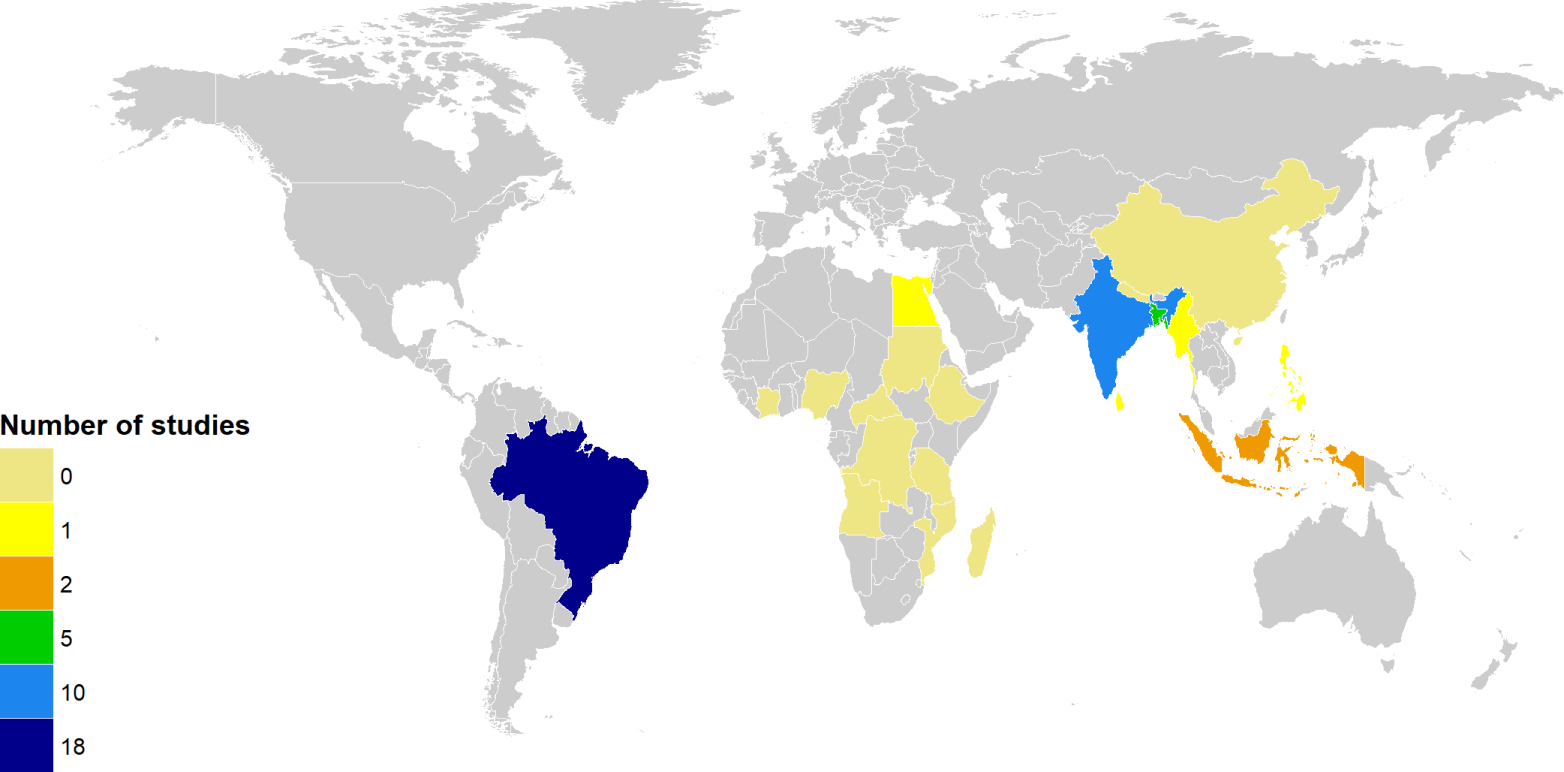
Number of eligible studies in countries officially reporting more than 1,000 cases per year in at least five consecutive or non-consecutive years between 2006 and 2016.

The protocol for the systematic review has been registered in the International Prospective Register of Systematic Reviews (PROSPERO) as CRD42016051212 [7]. To identify studies reporting associations between socioeconomic variables and leprosy outcomes in high-burden countries, we searched MEDLINE, Embase, LILACS, and Web of Science up to 20^th^ January 2017 using the strategy detailed in S1 Text and reviewed reference lists for additional relevant articles. No language restrictions were applied to the search; however, full text review was limited to articles published in English, Spanish, Portuguese, and French. Studies were eligible for inclusion if they: (i) were carried out in one of the 20 high-burden countries (i.e., defined as officially reporting more than 1,000 cases per year in at least five consecutive or non-consecutive years between 2006 and 2016 (Fig 1)[8, 9]; (ii) had a cohort, case-control, cross-sectional, or ecological study design; (iii) measured associations between one or more socioeconomic variables (i.e., age, sex, urban/rural residence, housing conditions/crowding, education/occupation, and social deprivation) and diagnosed leprosy disease. Studies were excluded if they: (i) had a qualitative or review design, (ii) exclusively used Phenolic Glycolipid I (PGL-1) positivity as a biomarker of leprosy exposure [10], (iii) lacked a clear description of the study population, or (iv) exclusively analyzed sex and/or age as the sociodemographic variables.

### Data extraction and analysis

Four reviewers (J.M.P, A.S., K.A., and L.M.S.) worked in duplicate to appraise records, evaluate study quality using the Newcastle-Ottawa scale (NOS) for individual level studies [11], and extract data using a standardized form (S1 Table). We used the NOS form for cohorts to evaluate data quality for cross-sectional studies; however the quality score was limited to a maximum of 7 points as it was not possible to demonstrate that leprosy was not present at the start of the study and due to the lack of follow up. Specifically, the reviewers extracted data related to the study protocol (i.e., geographic location, baseline survey dates, study design, study population, number of participants, method of leprosy ascertainment, and number of leprosy cases) and the measure of association (i.e., socioeconomic characteristics of leprosy cases and the comparison group, effect sizes, and statistical adjustment for potential confounders). Discrepancies were resolved by consensus. Individual level studies with data on different comparison groups (i.e., both cohort and case-controls in the same study) were considered in only one study, but data were extracted for all groups. Methods and results are reported following the Preferred Reporting Items for Systematic Reviews and Meta-Analyses (PRISMA) guidelines (for checklist, see S2 Table) [12].

The studies included in this review were summarized in two groups defined by whether the risk markers and leprosy outcomes were evaluated in individuals or at a population level. When estimates for a given risk marker was reported in at least three individualized studies, we estimated summary relative risks (RR) and its 95% Confidence Intervals (95% CI) by pooling effect sizes using random-effects meta-analyses. As leprosy is a rare disease, odds ratios and hazard ratios were assumed to approximate the same RR [13]. Studies conducted only among household contacts of leprosy patients or those with insufficient information to calculate the point estimates and its 95% CIs were not included in the meta-analysis. We assessed heterogeneity in RR estimates using *I*^*2*^ statistics and Cochran’s Q test *p*-values. Data analysis was performed in Stata, version 15.0, and R, version 3.4.0.

## Results

**Fig 2:**
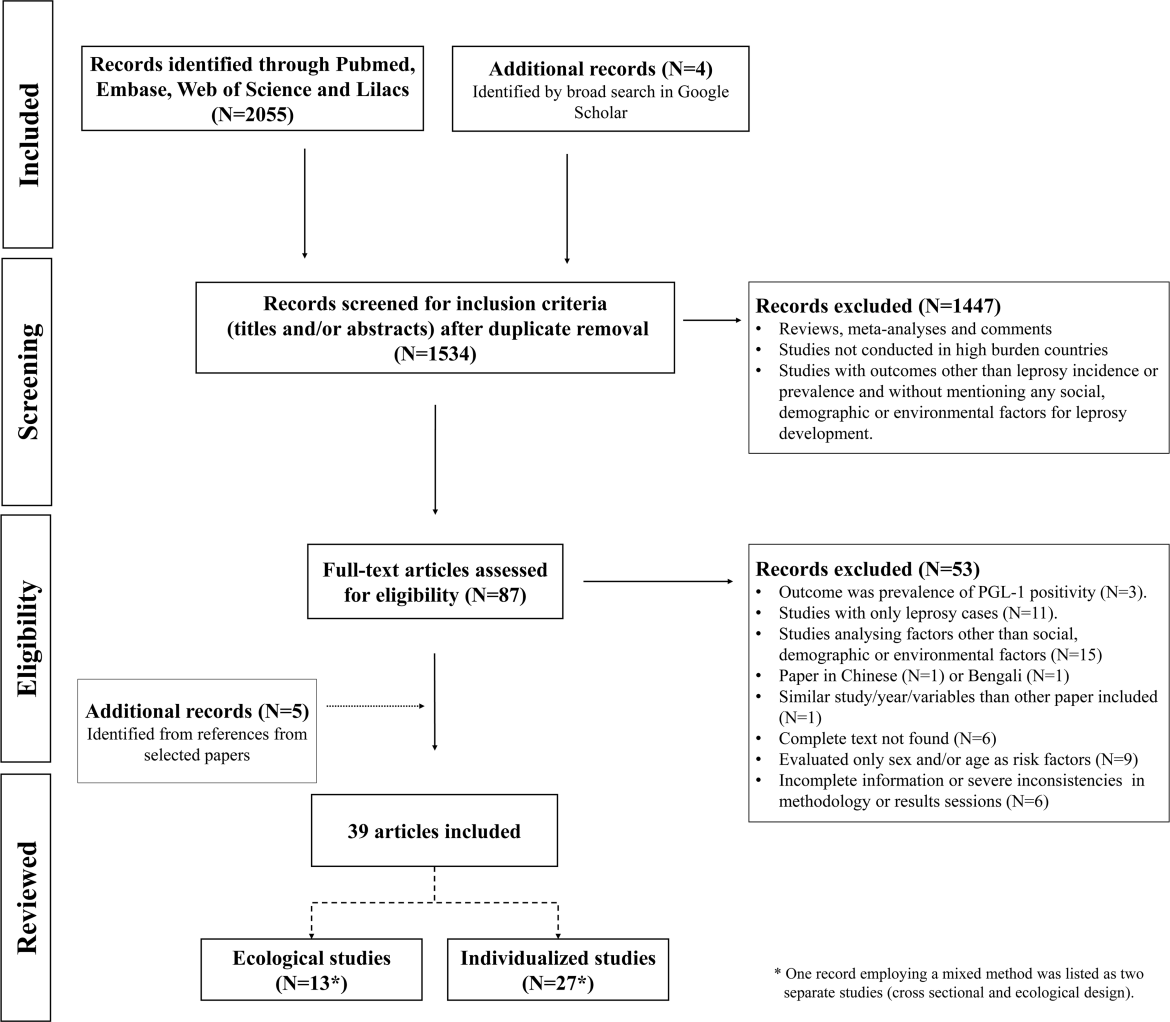
Flowchart for selection of studies.

The database search retrieved 1,534 independent records. After screening the abstracts, 96 full texts were reviewed, and 34 were selected for inclusion in the systematic review. Five additional eligible studies were identified through the references of the selected papers (Fig 2). Data were extracted from a total of 39 articles, comprising seven cohorts [14–20], seven case-controls [21–27], 13 cross-sectional studies [28–40], and 13 ecological studies [30, 41–52]; one record employing mixed methods (i.e., ecological and cross-sectional design) was listed as two separate studies (see Table 1 for individual studies and Table 2 for ecological studies). Of the individual studies, one cohort study assessed both the prevalence of leprosy in households containing an index case (cross-sectional) and followed those household contacts without leprosy prospectively [20]; a second study (case-control) considered two control groups, one proximal and one randomly selected [32].

**Table 1:** Observational studies conducted at the individual level of the association of leprosy with socioeconomic risk markers in high-burden countries.

Ref	Author (year)	Country	NOS	Study period	Type of study	Age	Total size	Leprosy cases	Frequency measure	Prevalence/ incidence in the studied area
**[14]**	Doull (1942)	Philippines	7	1936–37 (Talisay), 1933 (Cordova)	Cohort/Pop.	All ages	21,791	402	I	1/1,000 PYR (Talisay); 1/1,000 PYR (Cordova)
**[28]**	Nigam (1977)	India	6	1974–1975	Cross-sectional/ Pop.	All ages	3,362	18	P	5/1,000
**[29]**	Bhavsar (1980)	India	3	1976–1978	Cross-sectional/ Pop.	Children/Adolescents (5–19 years old)	21,412	26	P	12/10,000
**[15]**	Dominguez (1980)	Myanmar	6	1964–76	Cohort/ Pop.	All ages	52,026	1,367	I	NA
**[30]**	Sommerfelt (1985)	India	4	1982	Cross-sectional/ Pop.	All ages	7,428	131	P	18/1,000
**[31]**	Chaturvedi (1988)	India	4	1979–1983	Cross-sectional Pop.	All ages	63,321	691	P	11/1,000
**[21]**	George (1990)	India	8	1983–1984	Case-control/HB	All ages	288	72	-	NA
**[32]**	Andrade (1994)	Brazil	7	1988	Cross-sectional/ Pop.	All ages	926	137	P	NA
**[16]**	Ranade (1995)	India	9	1952–1886	Cohort/Contacts	Unspecified	6,284	331	I	5/1,000 PYR (24/1,000*)
**[33]**	Kumar (2001)	India	7	1999–2000	Cross-sectional/ Pop.	All ages	17,161	95	P	6/1,000
**[22]**	Bakker (2002)	Indonesia	6	June/July 2000 (1^st^ survey) and Nov 2000 (2nd survey)	Case-control/Contacts	Over 6 years old	192	96	P	195/10,000*
**[34]**	Hegazy (2002)	Egypt	5	1999–2001	Cross-sectional/ Pop.	All ages	9,643	24	P	25/10,000
**[35]**	Kumar (2003)	India	5	2000–2001	Cross-sectional/ Pop.	All ages	60,179	204	P	34/10,000
**[17]**	Bakker (2006)	Indonesia	7	2000–2004 (6 surveys)	Cohort/ Pop.	All ages	4,903	44	I	3/1,000 PYR
**[23]**	Kerr-Pontes (2006)	Brazil	5	2002	Case-control/ Pop.	Adults (>18 years old)	1,083	226	-	NA
**[36]**	Moet (2006)	Bangladesh	5	2002–2003	Cross-sectional/Contacts	Over 5 years old	21,870	159	P	7/1,000
**[18]**	Kumar (2007)	India	5	1999–2005	Cohort/ Pop.	All ages	42,113	77	I	6/10,000 PYR
**[19]**	Fischer (2008)	Bangladesh	7	1989–2003	Cohort/ Pop.	Unspecified	1,500,000**	11,060	I	1/1,000 PYR
**[37]**	Durães (2010)	Brazil	4	2004–2007	Cross-sectional/Contacts	All ages	1,040	211	P	NA
**[24]**	Feenstra (2011)	Bangladesh	8	2009	Case-control/ Pop.	Over 5 years old	289	90	-	NA
**[20]**	Sales (2011)	Brazil	8	1987 to 2007	Cohort and cross-sectional/Contacts	All ages	6,158	319 (133 new)	I	3/** PYR
**[25]**	Feenstra (2013)	Bangladesh	8	2009	Case-control/ Pop.	Over 5 years old	289	90	-	NA
**[38]**	Kumar (2013)	India	6	2009–2010	Cross-sectional/HB	All ages	804,536	355	P	4/10,000
**[39]**	Moura (2013)	Brazil	3	2006	Cross-sectional/Contacts	All ages	637	15	P	2/100
**[26]**	Murto (2013)	Brazil	5	2009–2010	Case-control/HB	Adults (>15 years old)	680	340	-	NA
**[27]**	Wagenaar (2015)	Bangladesh	7	2013	Case-control/ Pop.	Adults (18–50 years old)	152	52	-	NA
**[40]**	Dabrera (2016)	Sri Lanka	4	2012	Cross-sectional/ Pop.	All ages	753	39	P	511/10,000

Pop.: Population based; HB: Hospital-based; I: incidence; P: prevalence; PYR: person-years at risk; NA: not applicable.

*Prevalence in the survey that preceded the study.

** Denominator not specified.

**Table 2:** Ecological studies of the association of leprosy with socioeconomic risk markers in high-burden countries.

Ref	Author (year)	Country	Study period	Unit of analysis	Nº of study units	Leprosy cases	Frequency measure	Prevalence/ incidence in the studied area
**[30]**	Sommerfelt (1985)	India	1978 and 1982	Grouped villages	12	131	P	18/1,000
**[41]**	Kerr-Pontes (2004)	Brazil	1991–1999	Municipality	165	NR	I	1-15/10,000* (by municipality)
**[42]**	Lana (2009)	Brazil	2003–2006	Municipality	853	NR	I	NR
**[43]**	Imbiriba (2009)	Brazil	1998–2004	Census tracts	1,536	4,104	I	4/10,000*
**[44]**	Queiroz (2010)	Brazil	1995–2006	Census tracts	170	808	I	0-32/10,000* (by census tract)
**[45]**	Cury (2012)	Brazil	1998–2007	Census tracts	432	379	I	10/100,000
**[46]**	Barreto (2014)	Brazil	2004–2010	Census tracts	114	499	I	25-97/1000 (by census tracts)
**[47]**	Cabral-Miranda (2014)	Brazil	2005–2011	Municipality	417	1,674	I	1(2005) to 0.5/10,000 (2011)
**[48]**	Freitas (2014)	Brazil	2009–2011	Municipality	5,565	NR	I	9/100,000
**[49]**	Nery (2014)	Brazil	2004–2011	Municipality	1,358	200,966	I	75/100,000 (2004) to 46 /100,000 (2011)
**[50]**	Duarte-Cunha (2015)	Brazil	1998–2006	Neighbourhood	40	2,572	I	4/10,000
**[51]**	Nobre (2015)	Brazil	2001–2013	Municipality	167	3,927	I	8 (2001) to 9/100,000 (2013)
**[52]**	Castro (2016)	Brazil	2010	States	27	NR	I	22/100,000

P: Prevalence; I: incidence; NR: not reported.

*Yearly average new case detection rate in the study period.

The included studies were conducted in eight out of the 20 high-burden countries (Brazil [20, 23, 26, 32, 37, 39, 41–52], India [16, 18, 21, 28–31, 33, 35, 38], Bangladesh [19, 24, 25, 27, 36], Indonesia [17, 22], Egypt [34], Myanmar [15], Philippines [14] and Sri Lanka [40]—Fig 1). With the exception of Brazil, which is an upper-middle income country, all are classified as lower-middle income countries. The studies were published between 1942 and 2016, with the majority (N = 30) published after the year 2000. In the 31 studies that collected data from individual participants, prevalence estimates ranged from 12/10,000 persons in India [29] to 511/10,000 persons in Sri Lanka [40], while incidence estimates ranged from 0.49/1,000 person-years in Indonesia [19] to 2.88/1,000 person-years in Brazil [17] (see Table 1). The quality scores of the 27 individual level studies included varied across the study designs, with 11 studies receiving a score greater than or equal to seven (NB: NOS ranges from zero to nine). For the cohort studies, scores ranged from five to nine, and weaknesses were related to potential biases associated with loss to follow up. For the case-control studies, scores ranged from five to eight, with one study having a potential selection bias in the control group. For the cross-sectional studies, scores ranged from three to seven.

### Sex and age

**Fig 3:**
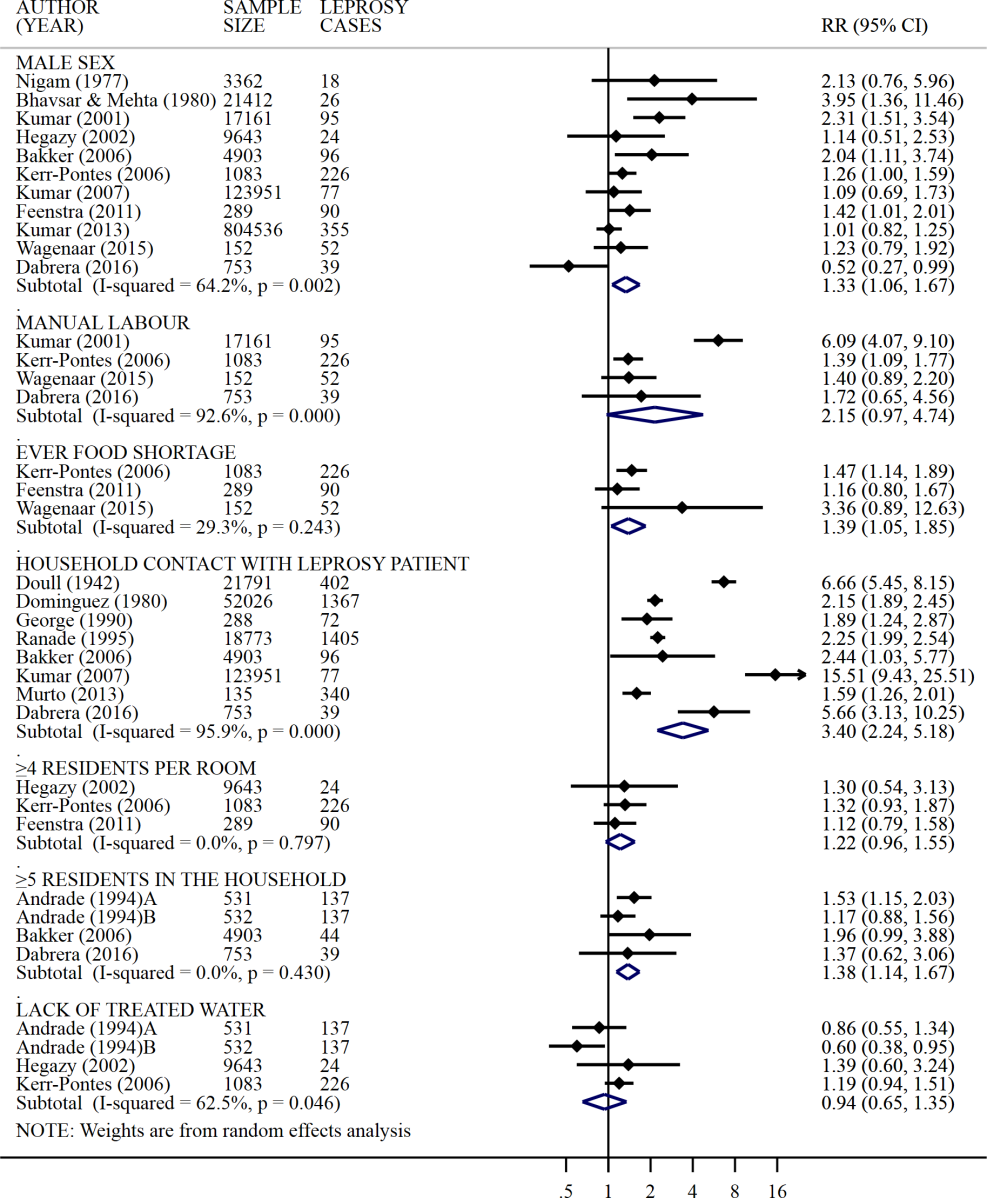
Association between leprosy and socioeconomic markers. Pooled estimates using random-effects meta-analyses are calculated by subgroups of socioeconomic variable. Error bars show the point RR with their 95% CIs on the log scale for each study. Diamonds show the combined point estimate. I^2^ statistic and Q-test *p*-value are reported.

Sex and/or age were investigated and/or adjusted for in 17 studies, including five cohorts [14, 16–18, 32], four case-controls [23, 24, 26, 27], and eight cross-sectional studies [29, 32–36, 38, 40]. Six out of 17 studies considered sex as a confounder in adjusted models, seven out of 13 considered age in the adjusted model, and five included both [20, 23, 26, 27, 33]. Fourteen studies analyzed the sex or age of the exposed and unexposed populations directly, one cross-sectional study examined the sex and age of family head [32], one cohort study evaluated the sex and age of the both the index patient and their contact [20], and one case-control study included sex and age only for adjustment without providing point estimates [26]. Out of 16 studies that investigated the association of leprosy with sex, four reported a higher prevalence of leprosy among males [14, 16, 17, 29], of which only one provided adjusted estimates. One study reported that contacts of male patients had higher leprosy incidence [20], and the others did not report differences between males and females. Eleven studies were included in the meta-analysis of the association between male sex and leprosy. The crude overall RR for male sex was 1.33 (95% CI: 1.06, 1.67), with a substantial heterogeneity between the studies (*I*^*2*^ = 64.2%) (Fig 3). The effect decreased along the study years. The association between age and leprosy was assessed in 13 studies, of which six found a positive association with increasing age [18, 24, 32, 34, 36].

### Education and occupation

The association between education and leprosy was evaluated in one cohort [20], three case-controls [23, 24, 26], and four cross-sectional studies [32–34, 40]. Different categorizations for education included family literacy [26], having formal education [33] and level of schooling [20, 23, 24, 32, 34, 40]. Three out of eight studies pointed to a higher number of leprosy cases among less educated individuals [23, 32, 33], and the associations remained significant after controlling for confounders (Table 3). In the study by Sales and colleagues, the educational level of the index patient was negatively associated with other prevalent leprosy cases within the family, but not among incident cases [20]. Andrade and colleagues (1994) suggested that a lower level of education was associated with higher leprosy incidence among neighbours, but not among other random groups [32]. Occupation status was analyzed in two case-controls studies [23, 27] and two cross-sectional studies [33, 40], most commonly by separating manual workers (e.g., factory, construction, or agriculture workers), from non-manual workers (e.g., traders or office workers) [23, 27, 33, 40]; unemployment as risk factor was also studied [40]. In the four studies included in the meta-analysis for occupation, there was a positive, but not statistically significant, association between leprosy and manual labor (RR = 2.15, 95% CI = 0.97–4.74; *I*^*2*^ = 92.6%) (Fig 3).

**Table 3:** Adjusted point estimates of the association of leprosy with socioeconomic risk markers in high-burden countries in individualized studies.

Ref	Year	Marker	Exposed group	Unexposed group	Type	Measure	Adjusted ^E^ for:				
							Sex	Age	Leprosy patient contact	Work or education	Others
**Education and occupation**											
**[32]**^A^	1994	**Education**	Less than High School	High School	ORadj	2.54 (1.06, 6.09)	□	■	□	□	■
**[32]**^B^	1994	**Education**	Less than High School	High School	ORadj	1.78 (0.79, 4.00)	□	■	□	□	■
**[33]**	2001	**Education**	No formal education	Formal education	ORadj	1.79 (1.11, 2.86)	■	■	□	■	■
**[23]**	2006	**Education**	Lower level of education	High level of education	ORadj	1.87 (1.29, 2.74)	■	■	□	□	■
**[20]**^D^	2011	**Education**	<4 years of formal education	>10 years of formal education	ORadj	0.82 (0.49, 1.36)	■	■	■	■	■
**[20]**^D^	2011	**Education**	<4 years of formal education	>10 years of formal education	ORadj	0.60 (0.34, 1.06)	■	■	■	■	■
**[20]**^C^	2011	**Education**	<4 years of formal education	>10 years of formal education	ORadj	1.43 (0.96, 2.15)	■	■	■	■	■
**[20]**^C^	2011	**Education**	<4 years of formal education	>10 years of formal education	ORadj	2.72 (1.54, 4.79)	■	■	■	■	■
**[33]**	2001	**Work type**	Housewives/students/others	Manual workers	ORadj	0.53 (0.28, 1.02)	■	■	□	■	■
**[27]**	2015	**Work type**	Business	Laborer	ORadj	0.66 (0.13, 3.25)	■	■	□	■	■
**Social deprivation and food security**											
**[23]**	2006	**Food availability**	Ever experienced food shortage	Never experienced food shortage	ORadj	1.54 (1.45, 1.63)	■	■	□	■	■
**[24]**	2011	**Food availability**	Food shortage in the past year	No recent food shortage	ORadj	1.79 (1.06, 3.02)	□	■	□	□	□
**[27]**	2015	**Food availability**	Household food stock present	Household food stock absent	ORadj	0.66 (0.29, 1.50)	■	■	□	■	■
**[27]**	2015	**Malnutrition**	Low diversity of food—Dietary Diversity Score ≤ 9	Higher diversity of food Dietary Diversity Score > 9	ORadj	0.83 (0.58, 1.18)	■	■	□	■	■
**Contact with leprosy patients**											
**[25]**	2013	**Contact**	Household contact	Social contacts outside the neighbourhood	ORadj	1.09 (1.01, 1.19)	□	■	□	□	■
**[25]**	2013	**Contact**	Social contacts within the neighbourhood	Social contacts outside the neighbourhood	ORadj	1.07 (1.03, 1.11)	□	■	□	□	■
**[36]**	2006	**Physical proximity (among contacts)**	Share the same roof and kitchen with a leprosy patient	Neighbors of next-door neighbors or social contacts	ORadj	2.44 (1.44, 4.12)	□	■	■	□	
**[20]**^C^	2011	**Physical proximity (among contacts)**	Household contact	Nonhousehold contact	ORadj	1.33 (1.02, 1.73)	■	■	□	■	■
**Living conditions and water supply**											
**[32]**^B^	1994	**Household construction**	Ground/cement floor	Carpet/wood/ceramic floor	ORadj	0.87 (0.49, 1.55)	□	■	□	■	■
**[32]**^B^	1994	**House ownership**	Non-private accommodation	House/flat	ORadj	3.95 (1.79, 8.72)	□	■	□	■	■
**[27]**	2015	**House ownership**	Landowner	Landless	ORadj	0.34 (0.14, 0.81)	■	■	□	■	■
**[32]**^A^	1994	**Household size**	Rooms in the household ≤ 2	Rooms in the household > 2	ORadj	0.76 (0.38, 1.53)	□	■	□	■	■
**[32]**^B^	1994	**Household size**	Rooms in the household ≤ 2	Rooms in the household > 2	ORadj	0.69 (0.45, 1.06)	□	■	□	■	■
**[27]**	2015	**Household size**	Household size (per m^2^)		ORadj	0.76 (0.55, 1.04)	■	■	□	■	■
**[32]**^B^	1994	**Clean water**	No tap water	Tap water	ORadj	0.37 (0.15, 0.91)	□	■	□	■	■
**[23]**	2006	**Clean water**	Regular bath in open waters in the past 10 years	No regular bath in open waters in the past 10 years	ORadj	1.77 (1.12, 2.81)	■	■	□	■	■
**[35]**	2003	**Sanitation**	Sanitary facility in the household	Household without a toilet	ORadj	1.39 (1.03, 1.89)	□	□	□	■	■
**[33]**	2001	**Household cleaniness**	Clean household	Dirty/very dirty household	ORadj	0.49 (0.33, 0.75)	■	■	□	■	■
**[35]**	2003	**Household cleaniness**	Clean household and surroundings	Dirty household and surroundings	ORadj	0.56 (0.36, 0.86)	■	■	□	■	■
**[23]**	2006	**Household cleaniness**	Low frequency of changing bed linen	High frequency of changing bed linen	ORadj	1.81 (1.30, 2.52)	■	■	□	■	■
**[17]**	2006	**Crowding**	Residents in the household ≥8	Residents in the household <8	HRadj	3.12 (1.34, 7.27)	□	□	□	□	■
**[20]**^C^	2011	**Crowding**	Residents in the household ≥5	Residents in the household <5	ORadj	0.71 (0.53, 0.95)	■	■	■	■	■
**[20]**^D^	2011	**Crowding**	Residents in the household ≥5	Residents in the household <5	ORadj	1.19 (0.79, 1.79)	■	■	■	■	■
**Other sociodemographic indicators**											
**[19]**	2008	**Health and social assistance**	Distance to health clinics (per 1 km)		RRadj	1.01 (0.98, 1.03)	□	□	□	□	■
**[27]**	2015	**Religion**	Hindu	Muslims	ORadj	1.41 (0.52, 3.88)	■	■	□	■	■
**[26]**	2013	**Migration**	Migrated in the past 5 year	Did not migrate in the past 5 years	ORadj	1.51 (1.0, 2.28)	■	■	■	■	■

^A^Households with leprosy patient compared with neighbor households.

^B^Households with leprosy patient compared with random household outside the neighborhood.

^C^Cross-sectional study assessing prevalence of leprosy inside the household with index leprosy case.

^D^Cohort study assessing the incidence.

^E^■ Presence or □ Absence

### Social deprivation and food security

The relationship between income and leprosy was assessed in one cohort [20], four case-controls [23, 24, 26, 27], and four cross-sectional studies [28, 29, 31, 34] using per capita household income [20, 26–29, 31] or socioeconomic position defined by self-assessment [27], assets score [24] or social score [34]). Three studies reported statistically significant associations between poverty and leprosy in univariate analysis [20, 27, 29], but the associations attenuated after adjusting for potential mediators, such as age, sex or occupation. Poverty measures differed among the studies, making a meta-analysis not appropriate; however, the direction of the association was consistent across studies, providing evidence of an inverse association between socioeconomic position and leprosy risk.

Factors related to food insecurity, an established correlate of poverty [53], were studied as a risk factor for leprosy in three case-control studies, two of which were carried out in Bangladesh [24, 27] and one in Brazil [23]. Food shortage in the past year was assessed twice [24, 27], ever food-shortage three times [23, 24, 27], and food expenditure, score of food insecurity (Household Food Insecurity Access Scale, HFIAS), Dietary Diversity Score (DDS), and household food stocks were evaluated once each [27]. Low food diversity and low stocks of food were not associated with increased number of leprosy cases, while food expenditure and HFIAS were negatively associated with leprosy [27]. In the meta-analysis, ever food-shortage was significantly associated with higher leprosy risks (RR = 1.39, 95% CI = 1.05–1.85; *I*^*2*^ = 29.3%) (Fig 3).

### Contact with leprosy patients

Sharing a household with a current leprosy case was strongly associated with risk of developing the disease in all nine studies that investigated this factor (five cohorts [14–18], three case-controls [21, 25, 26], and one cross-sectional study [40]). One study conducted by Feenstra and colleagues, which used a score of social interaction with a leprosy patient (i.e., in the household, within the neighborhood, and outside the neighborhood), found that contacts in the household and within the neighborhood shared similar risks of leprosy [25]. The meta-analysis of the other eight studies estimated a crude RR of 3.40 (95% CI = 2.24–5.18) associated with household sharing, with a substantial heterogeneity (*I*^*2*^ = 95.9%) (Fig 3). Six studies also evaluated the association between being a household or familial contact of a leprosy patient as opposed to any other type of contact, and all found that household or familial contacts had higher risk of leprosy than general contacts [16, 20, 22, 36, 37, 39].

### Living conditions and water supply

Household conditions were assessed in six studies, including three case-control and three cross-sectional studies, as house ownership [27], habitation type (i.e., private accommodation) [32], house size (i.e., in square meters and number of rooms) [24, 27, 32], and building or floor material [23, 31–33]. Neither owning the house [27], residing in private accommodation [32], nor house size [27] were significantly associated with leprosy after adjusting for factors such as education, work and household food stocks [27, 32]. Only one of the four studies looking at building materials found an association in univariate analysis between poorer building material (i.e., floor or house walls made of materials different than cement/bricks) and leprosy [31]. Crowding was measured as the number of residents in the household in four studies [17, 20, 32, 40] and residents per room in three studies [23, 24, 34]. Although only one individual study found evidence that crowding was significantly associated with higher leprosy risks [17], the pooled RR provides evidence that crowding, (i.e., ≥ five individuals living in the same household or ≥ four individuals sharing the same bedroom) may be a significant risk marker for leprosy (RR = 1.32, 95% CI = 1.13–1.53; *I*^*2*^ = 0.0%) (Fig 3). Of note, Kerr-Pontes and colleagues did not find an association between bed sharing and higher risk of leprosy [23].

Water and sanitation were investigated in one case-control [23] and in five cross-sectional studies [26, 29, 32, 34, 35]. Specifically, household access to clean water was assessed in three studies [23, 32, 34], waste collection in one [26], sanitation (sewage system or the presence of a sanitary facility in the house) in three studies, [23, 29, 35] and socio-sanitary score based on type of water supply and crowding in one [29]. Of the three studies investigating access to clean water, only the report by Andrade and colleagues found an association between clean water and a lower incidence of leprosy in adjusted estimates, when comparing households with leprosy with a random household, but not with a neighbouring household [32]. The presence of waste collection services [26] and good sanitary conditions score were associated with a lower prevalence of leprosy [29]. Cleanliness habits (e.g., sweeping the house, high frequency of changing bed linen) [23, 32] and household cleanliness (i.e., living in a dirty household or surroundings) [33, 35] were assessed in four studies, of which three found a negative association between cleanliness and leprosy [23, 33, 35]. Pooled statistics were calculated for lack of clean water in the household in three studies, including one with two comparisons group (RR = 0.94; 95% CI = 0.65,1.35; *I*^*2*^ = 62.5%) (Fig 3) and provided no evidence that clean water correlates with lower leprosy incidence.

### Other sociodemographic indicators

The studies at the individual level investigated a range of other sociodemographic factors, including ethnic background, marital status, religion, urbanization, and migration status, but the overall evidence was limited. For example, in the one case-control study that examined ethnicity and marriage as correlates of leprosy, the authors report no difference between white and black/brown or unmarried and married individuals [23]. The relationship between religion and leprosy was evaluated in three studies, one held in Bangladesh [27] and two in India [31, 33], with higher leprosy prevalence among Muslims reported in one [31]. In addition, of the three studies evaluating urbanicity and leprosy [29, 30, 38], two found that individuals living in urban (versus rural areas) [38] or in rural villages (versus the rural surrounding areas) have lower leprosy prevalence [30]. The distance from the household to health clinics, which can also be a measure of urbanization in mixed rural/urban areas, was evaluated by Fisher and colleagues (2008) in Bangladesh, but no relationship was found between leprosy detection rate and proximity to a clinic [19]. Recent migration (i.e., in the past 5 years) was evaluated once and was positively associated with leprosy [26].

### Ecological trends

Ecological studies provide an important line of evidence on the relationship between socioeconomic and demographic factors and leprosy (Tables 2 and 4). Associations of leprosy with increased urbanization [41, 45, 47–50], illiteracy/lower education [30, 41, 48–51] and unemployment [49–51] were consistently reported at the ecological level. Regions with a higher percentage of households with access to clean water [41, 50, 52], waste collection services [50, 51], or sanitation (i.e., a sewage system or a sanitary facility) [48, 50–52] reported a lower number of leprosy cases in the all but one of the studies [44, 48, 50, 52]. The mean number of individuals per household or per room was considered in seven studies [41, 46–50, 52], five of which found it positively associated with leprosy [46–49, 52]. Socioeconomic deprivation was measured as the percentage of people living in poverty or extreme poverty (i.e., according to a predefined threshold) [30, 41, 49–51], scores indicating poverty, socioeconomic groups, and social status (including deprivation) [43–45]. Half of these studies found a correlation between having better living conditions and lower leprosy burden [43–45, 49]. Migration, evaluated as the percentage of people born in other regions, was positively associated with leprosy [47]. Ecological studies also provided evidence of a correlation between malnutrition and leprosy among children [30, 51].

**Table 4:** Adjusted point estimates of the association of leprosy with socioeconomic risk markers in high burden countries in ecological studies.

Ref	Year	Marker	Exposed group	Unexposed group	Type	Measure
**Education and occupation**						
**[41]**	2004	**Education**	Children not going to school (per %)		βadj^1^	0.02 (0.00, 0.05)
**[41]**	2004	**Education**	Mean years of study among aged ≥ 25yrs (per year)		βadj^1^	1.35 (0.62, 2.08)
**[48]**	2014	**Education**	Illiteracy rate ≥ 24%	Illiteracy rate < 8%	RRadj	2.15 (1.83, 2.53)
**[49]**	2014	**Education**	Illiteracy rate ≥ 20.42%	Illiteracy rate < 20.42%	RRadj	1.12 (1.07, 1.18)
**[51]**	2015	**Education**	Illiteracy rate (per %)		ORadj	1.10 (0.98, 1.24)
**[49]**	2014	**Unemployment**	Unemployment rate ≥ 7.47%	Unemployment rate < 7.47%	RRadj	1.20 (1.16, 1.23)
**[51]**	2015	**Unemployment**	Unemployment rate (per %)		ORadj	1.03 (0.93, 1.14)
**Social deprivation and food security**						
**[49]**	2014	**Income**	Poor ≥ 27.42%	Poor < 27.42%	RRadj	1.13 (1.08, 1.18)
**[51]**	2015	**Income**	Per capita household income (per BRL)		ORadj	0.99 (0.98, 1.01)
**[51]**	2015	**Income**	Poor (<USD 70/month) (per %)		ORadj	0.94 (0.86, 1.03)
**[43]**	2009	**Economic and social indices/scores**	Low life conditions (index)	Fair life conditions (index)	ORadj	4.43 (3.14, 6.24)
**[51]**	2015	**Malnutrition**	Malnutrition in children <1 year old (per %)		ORadj	0.95 (0.62, 1.48)
**Living conditions**						
**[50]**	2015	**Clean water**	Households with water supply (per %)		RRadj	10.00 (2.32, 50.00)
**[48]**	2014	**Sanitation**	Households without adequate sanitation ≥ 16%	Households without adequate sanitation < 6%	RRadj	1.34 (1.47, 1.81)
**[51]**	2015	**Sanitation**	Households with adequate sanitation (per %)		ORadj	1.01 (0.98, 1.05)
**[51]**	2015	**Waste collection**	Households without adequate trash collection (per %)		ORadj	0.97 (0.92, 1.02)
**[47]**	2014	**Crowding**	Mean residents in the household (per unit)		RRadj	0.43 (*p* = 0.04)
**[49]**	2014	**Crowding**	Residents in the household ≥ 3.6	Residents in the household <3.6	RRadj	1.04 (1.01, 1.08)
**[48]**	2014	**Crowding**	Residents per room ≥ 0.65	Residents per room < 0.51	RRadj	1.41 (1.26, 1.58)
**Social and health indicators**						
**[49]**	2014	**Health and social assistance**	Coverage of Family Health Program > 95.06%	Coverage of Family health Program ≤ 72.02%	RRadj	1.12 (1.08, 1.17)
**[48]**	2014	**Health and social assistance**	Coverage of Family Health Program ≥ 80%	Coverage of Family health Program < 50%	RRadj	1.29 (1.17, 1.41)
**[50]**	2015	**Health and social assistance**	Number of health campaigns for leprosy detection (per unit)		RRadj	1.02 (0.96, 1.08)
**[50]**	2015	**Health and social assistance**	Number of reference units assisted by leprosy control programme (per unit)		RRadj	1.69 (1.10, 2.62)
**[51]**	2015	**Health and social assistance**	Vaccination coverage (per %)		ORadj	1.02 (0.95, 1.09)
**[49]**	2014	**Health and social assistance**	Coverage of cash transfer program ≥ 48.11%	Coverage of cash transfer program ≤ 27.75%	RRadj	0.79 (0.74, 0.83)
**[41]**	2004	**Inequality and human development**	Increased inequality (Theils L index) (per unit from 0 to 1)		βadj^1^	1.67 (0.39, 2.94)
**[49]**	2014	**Inequality and human development**	Inequality (Gini index) ≥ 0.54	Inequality (Gini index) < 0.54	RRadj	1.07 (1.04, 1.11)
**[48]**	2014	**Inequality and human development**	Inequality (Gini index) ≥ 0.55	Inequality (Gini index) < 0.50	RRadj	1.26 (1.16, 1.37)
**[47]**	2014	**Inequality and human development**	Increased inequality (Gini index) (per unit from 0 to 1)		RRadj	3.84 (*p* = 0.00)
**Population and environment**						
**[41]**	2004	**Urbanization**	Relative population growth between 1991 and 1999 (per %)		βadj^1^	1.02 (1.01, 1.04)
**[48]**	2014	**Urbanization**	Living in metropolis (municipality with > 900,000 inhabitants)	Living in small towns (municipality with up to 20,000 inhabitants)	RRadj	1.92 (1.15, 3.18)
**[48]**	2014	**Urbanization**	Urbanization rate ≥ 65%	Urbanization rate < 47%	RRadj	2.53 (1.40, 1.67)
**[49]**	2014	**Urbanization**	Urbanization rate ≥ 59.8%	Urbanization rate < 59.8%	RRadj	0.99 (0.93, 1.06)
**[49]**	2014	**Urbanization**	Urban population (per %)		RRadj	0.02 (*p*<0.01)
**[47]**	2014	**Migration**	Residents born in the State (per %)		RRadj	- 0.04 (*p* = 0.00)

^1^Linear regression.

Ecological evidence also suggests that, in general, indicators of social development and policy interventions were negatively associated with leprosy burden. Inequality was measured using Gini Index or Theil’s L index in four studies [41, 47–49] and as income ratio between the richest 20% and the poorest 20% (20–20 Income Ratio) in one study [48]. Human Development Index (HDI) was assessed in another study [42]. Overall, the studies provided strong and consistent evidence of an association between increased inequality and/or lower socioeconomic development and higher leprosy risks [41, 42, 47–49]. On the other hand, the presence of specific campaigns and health services for leprosy detection were associated with higher leprosy incidence rates, potentially by enhancing the leprosy detection efficiency [50]. While higher coverage of primary health care in Brazil was associated with higher leprosy new case detection in two studies [48, 49], no associations with leprosy were found using other metrics for health care access, including: the number of general public health services [41], number of physicians per 1,000 inhabitants [41], vaccination coverage [51] and infant mortality rates [41]. In Brazil, an analysis of the impact of a conditional cash transfer program showed that increased coverage of the program benefits was associated with a reduction in leprosy new case detection rates [49].

## Discussion

This systematic review points to a consistent relationship between leprosy and unfavorable socioeconomic circumstances. For individual level studies, meta-analyses provide evidence for increased risks of leprosy in individuals who are male, share homes with leprosy cases, live in crowded conditions, and have experienced food shortages in the past. In ecological level studies, point estimates for the associations between leprosy and sociodemographic risk markers of crowding, sanitation, and poverty remained largely consistent with individual level studies and across different geographic settings.

Overall, males had a greater risk of leprosy. However, the effect diminished in studies that are more recent; the pattern is potentially attributable to higher detection of leprosy among women over time and/or to change in exposure level of different risk markers in men and women. In most studies, literacy and high levels of education were associated with lower leprosy rates, although pooled estimates for education were not possible due to incomparable categories. Better education, in both sexes, can increase health knowledge and healthy behaviors, foster access to better work conditions and resources and promote greater autonomy [54], which could potentially reduce leprosy infection and transmission.

The type of work performed by an individual reflects their socioeconomic status and conditions and can vary across time and both within and between countries, especially in large and multicultural ones (e.g., India and Brazil). Pooled estimates between work and leprosy showed high statistical heterogeneity across the different studies, which might suggest that performing manual or agriculture work might correspond with different levels of poverty and living conditions in the different study settings (e.g., India, Brazil, Bangladesh or Sri Lanka), resulting in differences in the levels of exposure to *M*. *leprae* or chances of developing symptomatic disease. Food shortage, an indicator of extreme poverty and undernourishment [27] also appeared to be a risk marker of leprosy. Food-shortage was assessed in places where seasonality can influence work, income, food prices, consequently reducing dietary diversity [23, 24, 27]. More studies are needed about other possible risk markers of poverty and education inequalities, such as ethnicity [55, 56], which was assessed only once [23].

Person-to-person contact inside the household is one of the most likely sources for leprosy transmission [57]; nevertheless, similarities of social, sanitary, and poverty conditions shared by families and neighbors, which can contribute to leprosy transmission, are poorly taken into account. The higher leprosy prevalence among crowded households in the meta-analysis support the hypothesis that crowding can both facilitate transmission and also be a general indicator of poverty. Additionally, the association between religion and higher risk of leprosy in the study of Chaturvedi (1988) was mainly attributed to increased household crowding in some religious group [31], which also corroborates the idea that crowding may be associated with infection and/or disease development.

Most studies characterized the study setting as rural or urban areas, but only ecological studies showed consistent correlations between urbanization and higher leprosy rates. Studies performed at the individual level, showed that household characteristics and basic socio-sanitary conditions were strongly related with leprosy burden. In 2015, only 58% of the global population had access to clean water and 68% to adequate sanitation, with marked inequalities between rural/urban and rich/poor areas, including many high-burden countries for leprosy [58]. The absence of association between lack of access to clean water and leprosy in the meta-analysis might derive from high heterogeneity among the living conditions of those affected.

Migration from a relatively higher-burden setting is an important risk factor for infectious diseases transmission and reactivation in lower-burden settings (e.g., as has been previously demonstrated for tuberculosis) [59, 60]. This result differs from the two studies that evaluated migration history as a potential risk factor for leprosy. Nevertheless, the origin of migrants or the incidence/prevalence in their country or region of origin was not described.

The point estimates for the association between the socioeconomic or demographic characteristics (i.e., crowding, sanitation, and poverty) and leprosy in both individualized and ecological studies followed the same direction, suggesting no ecological fallacy and strengthening the association between these risk markers and leprosy. Nevertheless, it is important to mention that few studies reported the potential for reverse causality in both cross-sectional and ecological investigations (e.g., leprosy → unemployment). Freitas and colleagues (2014) suggested that higher detection rates of leprosy in municipalities with greater Family Health Program coverage can also be attributed to preferential targeting of municipalities by their leprosy rates [48]. Also, there is a possible link between leprosy-associated stigma and loss of employment, which could further worsen living conditions.

Some limitations of this systematic review include, first, the generalizability of the ecological findings as only one investigation was conducted outside of Brazil. Second, the findings presented here originate from studies carried out only in lower middle- and upper-middle economies, as we could not locate any relevant study carried out in a low-income country; the findings, although plausible, may be less applicable to low-income countries. Third, although we included a large number of social, demographic, and environmental factors as potential descriptors in the search strategy, some rare factors linked with leprosy burden might have missed. We selected all high burden countries for leprosy since 2001, but endemic countries facing civil war in the last 10 years might not have been included in WHO statistics or, by consequence, in this review. Fourth, heterogeneity of social/cultural/economic structures between countries and within large countries such as Brazil and India prevented us from combining characteristics such as education in the meta-analysis. Fifth, although the majority of studies were published in the 21^st^ century, the high-burden countries have experienced substantial economic growth in the past two decades, which has the potential to limit the generalizability of the meta-analysis estimates. Also, economic growth occurred in the past two decades, in which the majority of these studies have taken place could have contributed to higher heterogeneity in the effects between the studied social markers and leprosy. Despite these limitations, this review aggregated sparse evidence from diverse study settings, showing consistent associations between social determinants and leprosy across studies. Future research should prioritize investigations in low-income countries, address other markers of poverty (e.g., ethnicity, rural to urban migrants), explore heterogeneity between and within countries, and investigate the impact of recent poverty reduction programs.

Leprosy has been gradually included in the portfolio of diseases associated with poverty and in countries, like Brazil, has been incorporated into social programs [61]. For instance, high leprosy burden was accounted for in the prioritization of Brazilian municipalities in social protection programs, such as “Plano Brasil sem Miséria” [6]. Despite these advances, the options for combining curative approaches with prevention efforts particularly designed to address social determinants have not been fully considered in the context of leprosy control programs in many countries. Social determinants of leprosy have been poorly studied to date and need to be particularly addressed in those countries where leprosy incidence is still high and human development remains low. In agreement with the WHO Global Leprosy Strategy 2016–2020, which recommends the increase of inter-sectoral collaboration to further reduce the global and local leprosy burden, this review provides additional evidence that elimination of leprosy at the international level requires reduction of social inequalities, improving access of adequate housing and sanitation conditions and targeting social vulnerable groups and communities.

In conclusion, this study underscores the many ways that poverty can create conditions that perpetuate leprosy risk. In addition, these findings call attention to persistent gaps in knowledge of the associations between leprosy and socioeconomic risk markers and highlight a lack of studies conducted in low-income countries. Thus, political commitment must prioritize investments in not only the diagnosis of leprosy, but also in research on the social determinants of this ancient disease, and in the integration of leprosy-specific programs into social policies aiming to eradicate poverty.

## Supporting information

S1 Text Search strategy used to study the socioeconomic factors associated with leprosy burden. (DOCX)

S1 Table Summary table of the 39 appraised records. (PDF)

S2 Table Checklist for the PRISMA guidelines. (DOC)
