# Supplementary material for: Socioeconomic risk markers of leprosy in high-burden countries: A systematic review and meta-analysis
Source: PLoS Negl Trop Dis. 2018 Jul 9;12(7):e0006622. doi: 10.1371/journal.pntd.0006622 (PMC6053250; doi:10.1371/journal.pntd.0006622)
Supplement: S1 Text — (DOCX) [file pntd.0006622.s001.docx]

**S1 Text (Suppl 1)**

**Search strategy**

**Pubmed/medline**

1. Leprosy/epidemiology[MESH] OR LEPROSY[TIAB]
2. risk OR (social AND determinant*) OR demograph* OR economic* OR "living conditions" OR poverty OR poor OR unequal OR inequalit* OR socioeconomic* OR environment* OR "food shortage" OR "Risk Factors"[Mesh] OR "Socioeconomic Factors"[Mesh] OR "Demography"[Mesh]
3. epidemiolog* OR distribution OR incidence OR “detection rate” OR prevalence OR morbidity[MESH]
4. Angola* OR Bangladesh* OR Brazil* OR “Central African Republic” OR (central AND africa) OR China OR "Ivory Coast" OR "Democratic Republic of the Congo" OR Congo OR Egypt OR Ethiopia* OR India* OR Indonesia* OR Madagascar OR Myanmar OR Nepal OR Nigeria* OR Philippines OR "Sri Lanka" OR "United Republic of Tanzania" OR Tanzania OR Sudan OR Mozambique

**Embase**

1. 'leprosy'/exp/mj OR 'leprosy epidemiology'/exp/mj
2. risk OR 'social determinants of health' OR (social AND determinant*) OR demograph* OR economic* OR 'living conditions' OR poverty OR poor OR unequal OR inequalit* OR socioeconomic* OR environment* OR 'food deprivation' OR 'risk factor' OR socioeconomics OR 'demographic factors'
3. morbidity OR epidemiolog* OR distribution OR incidence OR 'detection rate' OR prevalence
4. angola* OR bangladesh* OR brazil* OR 'central african republic'/exp OR 'central african republic' OR (central AND ('africa'/exp OR africa)) OR 'china'/exp OR china OR 'ivory coast'/exp OR 'ivory coast' OR 'democratic republic of the congo'/exp OR 'democratic republic of the congo' OR 'congo'/exp OR congo OR 'egypt'/exp OR egypt OR ethiopia* OR india* OR indonesia* OR 'madagascar'/exp OR madagascar OR 'myanmar'/exp OR myanmar OR 'nepal'/exp OR nepal OR nigeria* OR 'philippines'/exp OR philippines OR 'sri lanka'/exp OR 'sri lanka' OR 'united republic of tanzania' OR 'tanzania'/exp OR tanzania OR 'sudan'/exp OR sudan OR 'mozambique'/exp OR Mozambique
5. AND ('article'/it OR 'article in press'/it OR 'conference abstract'/it OR 'conference paper'/it OR 'note'/it OR 'short survey'/it)

Resume of search strategy on Embase:

'leprosy'/exp/mj OR 'leprosy epidemiology'/exp/mj AND ('risk'/exp OR risk OR 'social determinants of health'/exp OR 'social determinants of health' OR (social AND determinant*) OR demograph* OR economic* OR 'living conditions' OR 'poverty'/exp OR poverty OR poor OR unequal OR inequalit* OR socioeconomic* OR environment* OR 'food deprivation'/exp OR 'food deprivation' OR 'risk factor'/exp OR 'risk factor' OR 'socioeconomics'/exp OR socioeconomics OR 'demographic factors') AND ('morbidity'/exp OR morbidity OR epidemiolog* OR distribution OR 'incidence'/exp OR incidence OR 'detection rate' OR 'prevalence'/exp OR prevalence) AND (angola* OR bangladesh* OR brazil* OR 'central african republic'/exp OR 'central african republic'OR (central AND ('africa'/exp OR africa)) OR 'china'/exp OR china OR 'ivory coast'/exp OR 'ivory coast' OR 'democratic republic of the congo'/exp OR 'democratic republic of the congo' OR 'congo'/exp OR congo OR 'egypt'/exp OR egypt OR ethiopia* OR india* OR indonesia* OR 'madagascar'/exp OR madagascar OR 'myanmar'/exp OR myanmar OR 'nepal'/exp OR nepal OR nigeria* OR 'philippines'/exp OR philippines OR 'sri lanka'/exp OR 'sri lanka' OR 'united republic of tanzania' OR 'tanzania'/exp OR tanzania OR 'sudan'/exp OR sudan OR 'mozambique'/exp OR mozambique) AND ('article'/it OR 'article in press'/it OR 'conference abstract'/it OR 'conference paper'/it OR 'note'/it OR 'short survey'/it)

**Lilacs**

1. leprosy OR lepra OR hanseniase OR lepra OR (mh:(lepra))
2. risk OR risco OR riesgo OR (socia* AND determinant*) OR demograph* OR demografic* OR economic* OR poverty OR poor OR pobre* OR unequal OR inequalit* OR desigual* OR iniquidad* OR socioeconomic* OR environment* OR "food deprivation" OR 'risk factor' OR socioeconomic* OR "demographic factors" OR ambienta*
3. morbidity OR morbidade OR epidemiolog* OR distribu* OR incidenc* OR "detection rate" OR prevalenc*
4. pais_assunto:("brasil")

Resume of search strategy on Lilacs:

(tw:((leprosy OR lepra OR hanseniase OR lepra OR (mh:(lepra))) )) AND (tw:((risk OR risco OR riesgo OR (socia* AND determinant*) OR demograph* OR demografic* OR economic* OR poverty OR poor OR pobre* OR unequal OR inequalit* OR desigual* OR iniquidad* OR socioeconomic* OR environment* OR "food deprivation" OR 'risk ractor' OR socioeconomic* OR "demographic factors" OR ambienta*))) AND (tw:(morbidity OR morbidade OR epidemiolog* OR distribu* OR incidenc* OR "detection rate" OR prevalenc*)) AND (instance:"regional") AND ( db:("LILACS") AND pais_assunto:("brasil"))

**Web of Science**

1. TS=leprosy
2. TS=(risk OR "social determinants of health" OR (social AND determinant*) OR demograph* OR economic* OR "living conditions" OR poverty OR poor OR unequal OR inequalit* OR socioeconomic* OR environment* OR 'food deprivation' OR "risk factor" OR socioeconomics OR "demographic factors")
3. TS=(morbidity OR epidemiolog* OR distribution OR incidence OR "detection rate" OR prevalence)
4. Refinado por: Países/Territórios: ( BRAZIL OR SRI LANKA OR INDIA OR ETHIOPIA OR MADAGASCAR OR BANGLADESH OR PEOPLES R CHINA OR EGYPT OR COTE IVOIRE OR NEPAL OR TANZANIA OR PHILIPPINES OR SUDAN OR MOZAMBIQUE OR NIGERIA OR INDONESIA)
