# Supplementary material for: Socioeconomic risk markers of leprosy in high-burden countries: A systematic review and meta-analysis
Source: PLoS Negl Trop Dis. 2018 Jul 9;12(7):e0006622. doi: 10.1371/journal.pntd.0006622 (PMC6053250; doi:10.1371/journal.pntd.0006622)
Supplement: S1 Table — (PDF) [file pntd.0006622.s002.pdf]

SUPPLEMENTARY FILE2. Table 2.1 Articles retained for the review

Ref	Author(s)	Country (further specifications, if any)	Study period (year)	Study design §	Unit of analysis §§	Short study description	Study population				Outcome			Confounders (N or list all, if any)	Social and/or demographic factors		Leprosy cases			Comparison group †††		Statistical analysis		Unadjusted association measure (95% CI or P-value)	Adjusted association measure (95% CI or P-value)
							Type of study §§§	Total size	Age	Study area †	Leprosy cases (No.)	Measure ††	Prevalence/ Incidence in the studied area		Factors studied	Categories	No. or mean	% or SD	Incidence or Prevalence (by category)	No. or mean	% or SD	Method #	Type of association measure ##		
(14)	Doull JA et al, 1942	Philippines (Talisay and Cordova municipalities)	1936-37 (Talisay), 1933 (Cordova)	CH	Individual	Retrospective study on contact status as a risk to develop leprosy.	PB	13,734 (217,729 PYR) in Talisay; 8,057 (117,287 PYR) in Cordova	<5 to 50+ years	NA	242 (Talisay), 160 (Cordova)	I	1.11 / 1,000 PYR (Talisay), 1.38 / 1,000 PYR (Cordova)	Sex	Household contact	No	252 (307,663 PYR)		0.82 / 1,000			Contingency table	RR‡		
																Yes	150 (27,353 PYR)		5.48 / 1,000					6.70‡ (5.43, 8.23)	
																No (male)			1.11 / 1,000						
																(female)			0.55 / 1,000						
																Yes (male)			6.69 / 1,000						
																(female)			3.87 / 1,000						
Incidence rate was over six times higher in household contacts (person who had lived under the same roof as a case for at least 1 month) than in non-contacts, and higher in males than females both in contacts and non-contacts																									
(28)	Nigam P et al, 1977	India (Bundelkhand area)	1974-1975	CS	Individual	Cross-sectional study carried out on the total population of three villages.	PB	3,362	All ages	RUR	18	P	5.35 / 1,000	N	Sex	M	13		7.03/ 1,000	1,835		Contingency table	OR‡	1.00	
																F	5		3.30/ 1,000	1,509				2.14 (0.71, 7.68)	
															Income level	≥Rupees 300 per capita/month	0			0				-	
																Rupees 50-299 per capita/month	0			38				-	
																Rupees 70-149 per capita/month	2		3.34/ 1,000	596				1.00	
																Rupees 30- 69 per capita/month	13		6.40/ 1,000	2,019				1.92 (0.43, 8.53)	
																<Rupees 30 per capita/month	3		4.32/ 1,000	691				1.29 (0.22, 7.77)	
																								1.21 (0.750) [1]	
																								0.10 (0.748) [2]	
Risk of leprosy was higher, although non significantly, in males, and in poorer people																									
(29)	Bhavsar BS et al,1980	India (Surat District)	1976-1978	CS	Individual	School survey.	PB	21,412	5-19 years	MX	26	P	12/10,000	N	Age	5 to 9 years	12		14/10,000	8,102		Contingency table	Chi square	1.54 (*p*>0.05)	
																10 to 14 years	11		9/10,000	11,665					
																15 to 19 years	3		81/10,000	1,645					
															Sex	Male	22		17/10,000	12,453				7.53*(*p*<0.01)	
																Female	4		4/10,000	8,959					
															Type of area	Urban	9		6/10,000	15,350				17.63*(*p*<0.001)	
																Rural	17		28/10,000	6,062					
															Social-economic groups	Class I	0		0	450				8.11*(*p*<0.05)	
																Class II	0		0	1,766					
																Class III	7		8/10,000	9,419					
																Class IV	16		19/10,000	8,595					
																Class V	3		46/10,000	650					
																missing	0		0	532					
															Score of socio-sanitary conditions of household	6 (best condition)	0		0	647				26.02*(*p*<0.001)	
																5	1		3/10,000	3,240					
																4	1		7/10,000	1,340					
																3	2		6/10,000	3,331					
																2	2		6/10,000	3,084					

[1] test of homogeneity (equal odds): chi square (*p*-value)
[2] score test for trend of odds: chi square (*p*-value)

																1	7		12/10,000	5,678					
																0	13		37/10,000	3,432					
																missing	0		0	660					
Prevalence of leprosy was lower than in other areas in India, but increased with deterioration of socioeconomic and home sanitary conditions.																									
(15)	Dominguez VM et al, 1980	Myanmar (Singu municipality)	1964-76: 1st survey in 1964-68, 2nd survey in 1969-72, annual follow-up of cases and contacts in 1965-76	CH	Individual	Estimation of incidence rates of leprosy in the 2nd mass survey among the individuals free of leprosy during the 1st survey (mean period four years) in 163 villages.	PB	52,026	All ages	NA	1,367	I	9.8/1,000 PYR (among contacts); 5.9/1,000 PYR (among non-contacts)	N	Household contact	No	1,090	2.3%	5.9‰ / year	45,446	97.7%	Contingency table	RR‡		
																Yes	277	5.1%	12.6‰ / year	5,203	94.9%			2.15 (1.89, 2.46)	
The mean yearly incidence rate in household contacts was over twice than in non-contacts																									
(30)	Sommerfelt H et al, 1985	India (North Arcot District)	1978 and 1982	CS + EC	Individual (CS); clusters (EC)	Two house-to-house surveys: one (1978) to assess demographic data and child nutritional status in 35 villages, the other one (1982) to assess leprosy prevalence in the the same villages. Correlation coefficients were used to assess the relationship between socio-demographic factors and leprosy incidence /1,000 inhabitants, among villages aggregated in 12 clusters.	PB	7,428 individuals; 12 clusters	All ages	RUR	131	P	17.9/1,000	N	CS study:										
															Type of area	Village	97		20.2/1000	4,702		Contingency table	Chi square with Yates correction	NA (*p*=0.03)	
																Field area	34		12.9*/1000	2,602					
															EC study:										
															Poverty (≤ 600 rupees)							Pearson correlation	R squared	0.099 (*p*=0.16)	
															% Illiteracy of all members of family									0.025 (*p*=0.31)	
															% Malnutrition in children 1-4 years old (mid-upper-arm circumference-MUAC)	MUAC<12.5 cm								0.318 (*p*=0.028)	
																MUAC<13.5 cm								0.410 (*p*=0.012)	
															% Malnutrition in general population									0.0055 (*p*=0.35)	
In villages/field areas and aggregates, lower leprosy prevalence rates was correlated to field areas. Moreover, there was a significant correlation between the occurrence of malnutrition in children 1 to 4 years of age and the prevalence of leprosy. Neither the occurrence of poverty nor illiteracy were correlated to leprosy prevalence.																									
(31)	Chaturvedi RM et al, 1988	India (Bombay)	1979-1983	CS	Individual	Data collected in Malwany suburb from different sources (mass house-to-house survey, school survey, household contact survey, clinical referral cases and self-reported cases) and analyzed all together.	PB	63,321	All ages	URB	691	P	10.91/1,000 (overall)	N	Religion	Hindu	331	47.90	9,17/1,000	36093	57.00	Difference in proportions	Z test	NA*(*p*<0.001)	
																Muslim	318	46.02	13,57/1,000	23428	37.0			NA*(*p*<0.05)	
																Christians	39	5.65	12,57/1,000	3102	4.9			NA (*p*=NS)	
																others	3	0.44	4,30/1,000	698	1.1			NA (*p*=NS)	
															Type of dwelling [3]	Zopadapatti	463	67.01	11,42/1,000	40557	64.05			NA*(*p*<0.001)	
																Chawls	221	31.98	10,11/1,000	21852	34.51			NA (*p*=NS)	
																Individual tenements	7	1.01	7,68/1,000	912	1.34			NA (*p*=NS)	
															Income per capita	0-50 rupees	327	47.32	20,82/1,000	15704	24.8			NA (*p*=NS)	
																51-100	283	40.96	10,16/1,000	27861	44.0			NA (*p*=NS)	
																101-250	70	10.13	4,57/1,000	15323	24.2			NA (*p*=NS)	
																>250	11	1.59	2,48/1,000	4433	7.0			NA (*p*=NS)	
															Monthly income (rupees)	mean	62			99.05				NA (*p*=NS)	
Leprosy is more prevalent among low socioeconomic status and overcrowded families. Prevalence is higher among Muslims, possibly because of overcrowding.																									

[3] Chawls: public housing buildings constructed between 1920 and 1956 by factory owners and landowners to accommodate migrant workers in India, consisting of one-room apartments with a small cooking space and common toilet facilities on each floor; zopadpattis: informal neighbourhoods created through ad-hoc construction techniques, representing the type of slum in India most commonly depicted by media.

(21)	George K et al, 1990	India (Asananbut village)	1983-1984	CC	Individual	Study on 72 cases from a SET[4] Unit and 216 matched controls from a Control Unit of the National Leprosy Eradication Programme.	HS	288	All ages	RUR	72	-	NA	N	Intrahousehold contact	No	53	21.9%	-	189	78.1%	Contingency table	OR	1.00	
																Yes	19	41.3%	-	27	58.7%			2.51* (1.23, 5.11)	
Household contacts had a higher risk of leprosy compared to non-contacts																									
(32)	Andrade VLG et al, 1994	Brazil (São Gonçalo municipality)	1988 (85 days)	CS	Domicile	Cross-sectional study to assess the household characteristics, the social and economic factors for leprosy transmission. A random sample, proportional to the number of leprosy cases in each census tract, was selected. Households with leprosy (group 1) were compared with one neighborhood group (group 2) and another random group (group 3).	PB	926	All ages	URB	137	P	NA	Group 1 vs Group 2: Education, age, time of residence in the household, number of rooms.	Type of housing	Aggregation	31			92		LR	OR	1.841 (0.825, 4.108)	
																House or flat	106			303				1.0	
															Size of household	≤ 50m2	79			259				0.888 (0.524, 1.505)	
																> 50m2	58			135				1.0	
															Household floor	Ground/cement	64			182				1.552 (0.930, 2.591)	
																Carpet, wood, ceramic	73			212				1.0	
															Water supply	No tap water	17			58				1.176 (0.567, 2.441)	
																Tap water	120			336				1.0	
															Number of rooms	≤ 2	42			184				0.589*(0.356, 0.964)	0.757 (0.376, 1.526)
																> 2	95			210				1.0	1.00
															Number of residents	≥ 5	63			127				1.351 (0.859, 2.126)	
																1 to 4	74			267				1.0	
															Sweep the house	Once a week	5			14				1.261 (0.404, 3.934)	
																Everyday	132			380				1.0	
															Age (head of the household)	> 40 years-old	99			185				4.326*(2.546, 7.352)	2.660*(1.321, 5.362)
																≤ 39 years-old	38			209				1.0	1.00
															Education (head of the household)	Primary/middle-school	123			305				2.707*(1.212, 6.046)	2.543*(1.063, 6.085)
																High-school	14			89				1.0	1.00
															Sex (head of the household)	Female	50			148				0.924 (0.593, 1.441)	
																Male	87			246				1.0	
															Time living in the address (head of the household)	>11 years	76			167				2.759*(1.610, 5.208)	1474 (0.714, 3.039)
																≤ 10 years	61			227				1.0	1.00
															Use of shoes (head of the household)	None or sandals	104			286				0.949 (0.504, 1.782)	
																Shoes	33			108				1.0	
														Group 1 vs Group 3: Education, age, time of residence in the household, type of housing, floor, water supply and number of rooms.	Type of housing	Aggregation	31			27				4.127*(2.344, 7.267)	3.950*(1.790, 8.717)
																House or flat	106			368				1.0	1.00
															Size of household	<=50m2	79			244				0.879 (0.563, 1.278)	
																>50m2	58			151				1.0	
															Household floor	Ground/cement	64			226				0.620*(0.414, 0.923)	0.872 (0.491, 1.548)
																Carpet, wood, ceramic	73			169				1.0	1.00
															Water supply	No tap water	17			85				0.421*(0.226, 0.787)	0.372*(0.151, 0.914)
																Tap water	120			310				1.0	1.00
															Number of rooms	<= 2	42			156				0.694 (0.453, 1.061)	0.694 (0.453, 1.061)
																> 2	95			239				1.0	1.00
															Number of residents	>= 5	63			161				1.243 (0.832, 1.856)	
																1 to 4	74			234				1.0	
															House sweeping	Once a week	5			18				0.861 (0.313, 2.370)	
																Everyday	132			377				1.0	
															Age (head of the household)	> 40 years-old	99			206				2.527*(1.601, 3.989)	2.071*(1.092, 3.927)
																<=39 years-old	38			189				1.0	1.00

[4] Survey, Education and Treatment

															Education (head of the household)	Primary/middle-school	123			313				2.106*(1.023, 4.350)	1.776 (0.788, 4.003)
																High-school	14			82				1.0	1.00
															Sex (head of the household)	Female	50			113				1.497 (0.985, 2.277)	
																Male	87			282				1.0	
															Time living in the address (head of the household)	>11 years	76			141				2.466*(1.510, 4.208)	1.519 (0.868, 2.659)
																<= 10 years	61			254				1.0	1.00
															Use of shoes (head of the household)	None or sandals	104			279				1.204 (0.698, 2.078)	
																Shoes	33			116				1.0	
Households, age and educational level are determinant factors for leprosy morbidity in this area. Comparing domiciles with leprosy (Group I) with their neighbors (Group II) and with domiciles outside the leprosy focus areas (Group III), the chance of a household have the disease when the head of the family have low educational level is 2.5 times higher than those with higher educational level when residing in the same area																									
(16)	Ranade MG et al, 1995	India (State of Maharashtra, 27 villages)	1952-1886	CH	Individual	Study on risk of leprosy among healthy contacts, based on annual surveys conducted in the area, covering about 85% of the population.	Contacts	12,489 (in 1952), 22,377 (in 1986), 6,284 healthy cont acts (overall)	NA (adult and children)	NA	331 (secondary cases), 1,074 (cases in non-contacts)	I	23.5 / 1,000 (prevalence in 1952); 4.46 / 1,000 PYR	N	Household contact	No	1,074		1.98/1,000	-		Contingency table	RR‡	1.00	
																Yes	331		4.46/1,000	5,953				2.26*(1.99, 2.55)	
															Closeness of contact (parent, child, grand parent, grandchild, spouse)	Not close (overall contacts)	109			2,166				1.00	
																Close	222			3,787				1.05 (0.96, 1.14)	
																Not close (male contacts)	73			1,085				1.00	
																Close	123			1,790				1.01 (0.90, 1.13)	
																Not close (female contacts)	36			1,081				1.00	
															Sex of contact	Close	99			1,997				1.13*(1.02, 1.26)	
																Female	135			3,078				1.00	
																Male	196			2,875				1.52*(1.23, 1.88)	
In the general population, risk of leprosy was over two times higher among household contacts than non-contacts; among contacts, chance of lepro sy was higher among males than females, but closeness of contact was a significant risk only in female contacts																									
(33)	Kumar A et al, 2001	India (Agra district)	1999-2000	CS	Individual	One year survey in domiciles and schools in each of the 90 villages/urban units from Agra, aiming to study the persistently high case detection remain in the region and the socio-demographic factors related to leprosy prevalence.	PB	17,161 (13,320 rural and 3,841 urban)	<15to 69+ years	MX	95	P	5.5/1,000; 1.25/1,000 (in <15 year olds)	Age, sex, area	Area[5]	Urban	15		3.9/1,000	13,320		LR	OR (combining data from rural and urban areas)	-	-
																Rural	80		6.0/1,000	3,841				-	-
															House cleanliness	No	-			-				1.00	1.00
																Yes	-			-				0.48*(0.32, 0.73)	0.49*(0.33, 0.75)
															Formal education	No	-			-				1.00	1.00
																Yes	-			-				0.95 (0.61, 1.49)	0.56*(0.35, 0.90)
															Occupation	Blue collar	-			-				1.00	1.00
																Housewifes/students/others	-			-				0.45*(0.29, 0.71)	00.53*(0.28, 1.02)
															Rural area:										
															Age	Under 15 years	8		1.3/1,000	6,494					
																15 to 29	11		3.8/1,000	2,915					
																30 to 44	16		8.3/1,000	1,939					
																45 to 59	21		18.6/1,000	1,131					
																59 or more	24		28.5/1,000	841					
															Sex	Male	56		8.9/1,000	6,310					
																Female	24		3.4/1,000	7,010					
															Education	None	53		7.3/1,000	7,270					
																Formal	27		4.5/1,000*	6,050					
																<=5 years	10		3.0/1,000	3,348					
																5-10 years	15		6.7/1,000	2,251					
																>10 years	2		4.4/1,000	451					
															Occupation	Agriculture/blue collar	44		19.7/1,000*	2,230					
																Others	36		3.3/1,000	1,090					
																none	12		3.1/1,000	3,817					
																students	6		1.6/1,000	3,678					
																housewifes	18		5.0/1,000	3,595					
															Religion	Hindu	78		6.7/1,000	11,565					

[5] Rural and urban areas corresponded to different surveys

																Muslin	1		2.5/1,000	404					
															House type	Kuccha (mud and straw)	40		7.8/1,000	5,126					
																Pucca (cement/briks)/Semi-pucca(mixed)	39		5.4/1,000	7,289					
																Clean	37		4.9/1,000	7,505					
															House cleanliness	Dirty/very dirty	42		9.4/1,000	4,464					
																Clean	25		5.1/1,000	4,883					
															Surroundings	Dirty	54		7.6/1,000	7,086					
															Urban area:	Under 15 years	2		1.1/1,000	1,880					
															Age	15 to 29	3		3.2/1,000	942					
																30 to 44	7		1.2/1,000	584					
																45 to 59	2		7.6/1,000	263					
																59 or more	1		5.8/1,000	172					
																Male	8		4.5/1,000	1,790					
															Sex	Female	7		3.4/1,000	2,051					
																None	9		5.1/1,000	1,776					
															Education	Formal	6		2.9/1,000	2,065					
																<=5 years	2		2.5/1,000	801					
																5-10 years	4		3.8/1,000	1,062					
																>10 years	0		0.0/1,000	202					
																Agriculture/blue collar	8		13.0/1,000*	614					
															Occupation	Others	7		2.3/1,000	3,227					
																none	1		1.1/1,000	918					
																students	1		0.8/1,000	1257					
																H/W	5		4.8/1,000	1,052					
																Hindu	12		3.5/1,000	3,460					
															Religion	Muslin	3		7.9/1,000	380					
																Kuccha (mud and straw)	2		7.8/1,000	258					
															House type	Pucca (cement/briks) / Semi-pucca (mixed)	13		3.6/1000	3,578					
																Clean									
																Dirty/very dirty	6		2.1/1,000	2,833					
															House cleanliness	Clean	9		8.9/1,000*	1,008					
																Dirty	2		1.8/1,000	1,105					
															Surroundings		13		4.8/1,000	2,736					
The study found a higher prevalence of leprosy in rural areas. Formal education and living in clean housing was associated to lower risk of leprosy. Agricultural/blue collar workers, persons living in dirty surroundings and persons living in houses with insufficient exposure to sunlight also have a higher risk of leprosy.																									
(22)	Bakker MI et al, 2002	Indonesia (Tampaang, Pelokang, Kembanglemari, Sailus and Sapuka)	June/July 2000 (1st survey) and Nov 2000 (2nd survey)	CC	Individual	Case-control study on the clustering of leprosy cases by type of contact in five islands.	Contacts	4,140	6 to 73 years	NA	96 (85 new cases, 11 old cases)	I<br>P [6]	205 (95% CI 162, 248) /10,000<br>195 ((95% CI 156, 234)/10,000	N	Type of clustering of patients and controls	House<br>House+N1 (N1, direct neighbors)<br>House+N1+N2 (N2, next neighbors)	NA	32%<br>52%<br>64%	NA	NA	16%<br>30%<br>47%	Chi square	Patients/ controls rate	2.07 (0.007)<br>1.75 (0.002)<br>1.34 (0.029)	
For all three types of clusters (with at least another patient), the proportion of patients clustered was higher than the proportion caused by chance, and the effect diminished when widening the cluster																									
(34)	Hegazy AA et al, 2002	Egypt (Kafr-Tambul village)	1999-2001	CS	Individual	Household survey	PB	9,643	All ages	RUR	24 (5 new cases,19 old cases)	P	24.9 (95% CI 16.3, 37.6) / 10,000	N	Age	<20 years<br>20-30<br>30-40	4<br>5<br>5	0.1%<br>0.3%<br>0.3%	0.99/1,000<br>3.11/1,000<br>3.30/1,000	4,005<br>1,600<br>1,510	99.9%<br>99.7%<br>99.7%	LR	OR	1.00<br>3.13 (0.73, 1.74)<br>3.32 (0.78, 1.54)	

[6] cases registered for therapy at the end of the 2nd survey

																40+	10	0.4%	3.97/1,000	2,504	99.7%			4.00 (1.16, 1.54)	
															Sex	Female	11	0.2%	2.33/1,000	4,714	99.8%			1.00	
																Male	13	0.3%	2.64/1,000	4,905	99.7%			1.14 (0.48, 2.41)	
															Education	Secondary and higher	2	0.1%	1.01/1,000	1,982	99.9%			1.00	
																Primary and preparatory	4	0.1%	1.43/1,000	2,802	99.9%			1.41 (0.22,11.09)	
																Illiterate/reads and writes	18	0.4%	3.71/1,000	4,835	99.6%			3.69 (0.83,23.02)	
															Crowding	<4 persons/room	17	0.2%	2.32/1,000	7,308	99.8%			1.00	
																≥4 persons/room	7	0.3%	3.02/1,000	2,311	99.7%			1.30 (0.49, 3.33)	
															Water supply	Piped water	8	0.2%	2.02/1,000	3,946	99.8%			1.00	
																Hand pump	16	0.3%	2.81/1,000	5,673	99.7%			1.39 (0.56, 3.54)	
															Social economic positionscore	Moderate to high	5	0.1%	1.33/1,000	3,756	99.9%			1.00	
																Low	19	0.3%	3.23/1,000	5,863	99.7%			2.43 (0.86, 7.44)	
Risk of leprosy was significantly higher in individuals older than 40 years; the association of leprosy with male sex, low degree of education, crowding, unhealthy source of water and low social score was not significant																									
(35)	Kumar A et al, 2003	India (Agra district)	2000-2001	CS	Individual	Door-to door survey conducted during one year in the whole urban area in Agra district.	PB	60,179	All ages	URB	204	P	33.9 (95% CI 9.7-40.7) /10,000	Type of housing, sex, age.	Age	<15 years	12		4.4/10,000	27,566		Contingency table/ LR	Chi square / OR		
																> 15 years	192		58.9/10,000	32,409					
															Sex (among >15 years old)	Men > 15 years			92.0/10,000*						
																Woman > 15 years			41.6/10,000*						
															Cleanliness of the house	Slovenly houses/dirty surroundings			64.3/10,000*					10.9*(p<0,0001)	1.00
																Clean houses/clean surroundings			31.9/10,000*						0.56*(0.36, 0.86)
															Work	Manual workers			94.9/10,000*					137.5*(p<0,0001)	1.00
																Others			21.3/10,000*						0.63*(0.43, 0.95)
															Toilet in the household	No			39.1/10,000*					5.6*(p = 0,015)	1.00
																Yes			25.5/10,000*						0.72*(0.53, 0.97)
Higher leprosy prevalence was observe among adults and males. Living in bad sanitary conditions was also associated to increased risk of having leprosy.																									
(41)	Kerr-Pontes LRS et al, 2004	Brazil (State of Ceara, 165 muni cipalities)	1991-1999	EC	Municipality	Ecological study on the association of leprosy incidence rate with selected socioeconomic and environmental factors.	PB	165	<15 years	NA	NA	I[7]	0.06 to 14.68 / 10,000 (municipalities yearly average rates over 1991-1999)	Level of inequality[8]	Level of inequality		NA	NA	NA	NA	NA	LN	Pearson correlation (univariate) and β coefficients (95% CI)	0.24 (p<0.05)	1.67 (0.389, 2.944)
														Mean years of study among aged ≥25yrs	Mean years of study among aged ≥25yrs									0.32 (p<0.01)	1.35 (0.620, 2.081)
														% population growth in 1991-1996	% population growth in 1991-96									0.16 (p<0.05)	0.02 (0.006, 0.038)
														% children 7-14 years old not going to school	% children 7-14 years old not going to school									-0.09 (p=NSs)	0.02 (0.003, 0.045)
														Presence of railroad (Y)	Presence of railroad (Y)									NA	0.45 (0.025, 0.871)
															% households with public water supply									0.13 (p=NS)	
															Infant mortality rate									0.09 (p=NS)	
															Public Health Services per 1000 inhabitants									0.13 (p=NS)	
															Physicians per 1000 inhabitants									0.32 (p<0.01)	
															% households with density >2 people per room									0.01 (p=NS)	
															% head of family with monthly income ≤ half the minimum wage									-0.14 (p=NS)	
Average incidence rate of leprosy was positively associated with a higher degree of inequality and a higher % of children 7–14 years old not going to school. The presence of railroad, a higher population growth in 1991-1996 and more years of study among 25 year-old people, which are collectively interpreted as indicators of a rapid and uncontrolled urbanization, were also associated with leprosy.																									

[7] Yearly average incidence rate 1991-99 (cases/ 10,000)
[8] Theil's L Index

(17)	Bakker MI et al, 2006	Indonesia (five islands: Tampaang, Pelokang, Kembanglemari, Sailus and Sapuka)	2000-2004 (6 surveys)	CH	Individual	Cohort study on factors associated with leprosy incidence, as part of a study on the impact of rifampicin prophylaxis on the disease incidence.	PB	4,903 (177,569 person-months)	All ages	NA	44	I	2.98 (95% CI 2.2, 4.0) / 1,000 PYR	(A): sex, household size, serological status in 2000 and contact status by classification of index patient, adjusted for each other + intervention (rifampicin chemoprophylaxis)	Sex	Female	17	0.7%	2.04 (1.27, 3.29)	2,594	99.3%	CX	HR	1.0	1.0 [12]
																Male	27	1.2%	4.17 (2.86, 6.08)	2,265	98.8%			2.01 (1.10, 3.70)	2.21 (1.20, 4.09)
															Age	0–5	1		0.47 (0·07, 3.33)					0.13* (0.02, 0.95)	
																6–14	14		3.70 (2.19, 6·24)					1·0	
																15–29	14		3.53 (2.09, 5·95)					0.92 (0·44, 1.94)	
																30–44	10		3.81 (2.05, 7·08)					1.01 (0·45, 2.28)	
																45–59	3		2.16 (0.70, 6·70)					0.55 (0·16, 1.92)	
																>60	2		2.24 (0.56, 8·96)					0.57 (0·13, 2.52)	
															Household members	1-4	11	0.6%	1.88 (1.04, 3.40)	1,917	99.4%			1.0 [11]	1.0 [12]
																5-7	21	0.9%	3.09 (2.02, 4.74)	2,229	99.1%			1.71 (0.82, 3.56)	1.61 (0.77, 3.37)
																8-16	12	1.7%	5.61 (3.19, 9.88)	713	98.3%			3.47 (1.51, 7.98)	3.12 (1.34, 7.27)
															Contact status 1	No contact [9]	29		2.88 (2.00, 4.15)					1.0 [11]	
																N2 contact	6	0.7%	3.31 (1.49, 7.38)	4,581	99.3%			1.52 (0.50, 4.59)	
																N1 contact	3		1.48 (0.48, 4.60)					0.72 (0.19, 2.75)	
																Household contact	6	2.1%	6.67 (3.00, 14.9)	278	97.9%			3.29 (1.11, 9.77)	
															Contact status 2	No contact (>100m) [10]	15		2.69 (1.62, 4.46)					1.0 [11]	
																Buffer contact 75-100 m	3		1.75 (0.56, 5.43)					0.62 (0.18, 2.15)	
																Buffer contact 50-75 m	8		4.01 (2.00, 8.01)					1.51 (0.64, 3.56)	
																Buffer contact 25-50 m	7		3.15 (1.50, 6.60)					1.33 (0.52, 3.42)	
																Buffer contact 1-25 m	5		2.10 (0.87, 5.04)					1.15 (1.36, 3.62)	
																Household contact	6		6.67 (3.00, 14.2)					3.57 (1.18, 10.7)	
The risk among males to develop leprosy was over twice higher than among females. The risk among household contacts was over three times higher in households with more than 7 members compared to 1-4 member households.																									
(23)	Kerr-Pontes LRS et al, 2006	Brazil (State of Ceará)	2002	CC	Individual	Study in four municipalities aimed to identify socioeconomic, environmental, and behavioral factors associated with leprosy occurrence in individuals with no contact with leprosy patients. For each case, four age and sex frequency matched individuals, presenting for reasons other than skin problems to the health unit where the case was diagnosed and living in the same municipality as the case, were selected as controls. For multivariate analysis, a hierarchical framework in five blocks (block 1: socio economic factors; block 2: environmental factors; block	PB	1,083	>18 years	NA	222	-	NA	Bivariate analysis was done accounting for cluster effect of municipalities; multivariate analyses were done in each block, adjusting for all variables in the block (first step), and then, adjusting the socioeconomic block by the statistically significant variables of the four blocks left (second step, shown here)	Block 1:							LR	OR		
															Schooling	High	40	18		244	28			1.00	1.00
																Middle	56	25		226	26			1.51 (0.93, 2.47)	1.50 (0.91, 2.50)
																Low	130	58		387	45			2.05*(1.29, 3.27)	1.87*(1.29, 2.74)
															Food shortage at any time in life	Never experienced	161	72		687	81			1.00	1.00
																Experienced	63	28		163	19			1.65*(1.11, 2.42)	1.54*(1.45, 1.63)
															Access to safe drinking water in the past 10 years	Yes	133	59		546	65			1.00	
																No	91	41		298	35			1.17 (0.96, 1.43)	
															Sewage disposal in the past 10 years	Yes	180	81		741	87			1.00	
																No	41	19		111	13			1.44 (0.95, 2.80)	
															Sand/mud in the floor in the past 10 years	No	182	81		737	86			1.00	
																Yes	44	20		119	14			1.46*(1.04, 2.06)	
															Block 2:										
															Household crowding (currently)	0–3 persons per room	200	89		782	92			1.00	
																4 or more persons per room	26	12		71	8			1.43 (0.64, 3.20)	
															Has/had animals in the house/yard in the past 10 years	No	39	17		203	24			1.00	
																Yes	184	83		649	77			1.48 (0.77, 2.86)	
															Works/worked in forest in the past 10 years	No	148	69		647	76			1.00	
																Yes	68	31		200	24			1.43 (0.90, 2.29)	
															Works/worked in agricultural field in the past 10 years	No	81	36		392	46			1.00	
																Yes	144	64		463	54			1.48 (0.79, 2.77)	
															Block 3:										

[9] reference category for contacts in household, N1 (direct neighbours) and N2 (next neighbours)
[10] reference category for contact classification by spatially defined buffers
[11] adjusted by intervention
[12] adjusted by (A) (see list of confounders)

						3: behavioral factors; block 4: demographic factors and block 5: vaccination) was defined.									Frequency of changing bed linen (current)	Biweekly	132	58		609	72			1.00	1.00
																>Biweekly	94	42		242	28			1.79* (1.32, 2.43)	1.81* (1.30, 2.52)
															Sharing its own bed/hammock with others (current)	Yes	100	44		428	50			1.00	
																No	125	56		426	50			1.29 (0.93, 1.61)	
															Sharing others bed/hammock with others (current)	Yes	131	58		526	62			1.00	
																No	95	42		322	38			1.17 (0.60, 2.30)	
															Weekly regular bath in open water bodies (creek, river or lake) in the past 10 years	No	188	83		770	90			1.00	1.00
																Yes	38	17		87	10			1.79*(1.18, 2.70)	1.77* (1.12, 2.81)
															Block 4:										
															Sex	Male	108	48		348	41			1.00	1.00
																Female	118	52		509	59			0.84 (0.68, 1.04)	0.97 (0.70, 1.34)
															Age (years) - continuous in the multivariable analysis	< 30	44	20		228	27			1.00	1.01 (1.00, 1.02)
																30–39	33	15		167	19			1.02 (0.72, 1.45)	
																>40	149	66		462	54			1.67 (0.77, 3.64)	
															Skin colour	White	76	34		404	47			1.00	
																Brown/black	148	66		450	53			1.88 (0.99, 3.56)	
															Marital status	Not married	78	35		298	35			1.00	
																Married	144	65		552	65			0.95 (0.84, 1.06)	
Crowding or sharing the bed or hammock with other household members did not show a significant association with leprosy. Low education level, ever having experienced food shortage, bathing weekly in open water bodies (creek, river and/or lake) 10 years previously, and a low frequency of changing bed linen or hammock (>biweekly) currently were all associated with leprosy.																									
(36)	Moet FJ et al, 2006	Bangladesh (Districts of Nilphamari and Rangpur)	2002-2003	CS	Individual	Study on prevalence of leprosy among contacts of leprosy patients and its association with different characteristics in contacts and patients (part of COLEP project)	Contacts	21,870 contacts	5 to 50+ years	RUR	159	P	7.3 (95% CI 6.2,8.5) / 1,000	Age, type of leprosy, physical distance, genetic distance	Sex	Female	NA		-	NA	NA	LR	OR	1.00	
																Male			-					1.26 (0.92, 1.72)	
															Age, years	5- 9			3.3 (1.7, 5.9) [14]					1.00	1.00
																10-14			6.5 (4.1, 9.7) [14]					1.97 (0.96, 4.04)	2.02 (0.98, 4.15)
																15-19			9.8 (6.3,14.5) [14]					2.98 (1.46, 6.09)	3.08 (1.49, 6.34)
																20-29			5.0 (3.0, 7.9) [14]					1.53 (0.73, 3.22)	1.72 (0.81, 3.63)
																>30			9.3 (7.4,11.6) [14]					2.84 (1.51, 5.34)	2.94 (1.56, 5.54)
															Physical distance [13]	N2+S			4.9 (3.8, 6.3) [14]					1.00	1.00
																R+N1			8.7 (6.5,11.5) [14]					1.79 (1.23, 2.60)	1.69 (1.16, 2.47)
																K			7.5 (3.9,13.1) [14]					1.54 (0.83, 2.87)	1.05 (0.52, 2.13)
																KR			15.6 (10.6,22.0) [14]					3.21 (2.08, 4.96)	2.44 (1.44, 4.12)
In this study, the contacts living under the same roof as patients and sharing the same kitchen had a higher risk than other contacts living under the same roof and next door neighbors, who, again, had a higher risk than neighbors of neighbors.																									
(18)	Kumar A et al, 2007	India	1999-2005	CH	Individual	Study on the leprosy-free population (in a survey conducted in 1999 to 2001), resurveyed from 2 to over 4 years after the initial survey.	PB	42,113 / 123,951.2 PYR	<15 to 44+ years	MX	77	I	6.2/10,000 overall; 4.5/10,000 in <15years old	No	Contact with leprosy patient	non-familial contacts	56		4.6/10,000	41,119			RR	NA (p<0.01)	
																familial contacts	21		67.6/10,000	994					
															Age	< 15	25		4.5/10,000	18,745				NA (p<0.005)	
																15 to 29	15		5.2/10,000	9,857					
																30 to 44	17		7.8/10,000	7,446					
															Sex	44 or more	20		11.2/10,000	6,065					
																Males	29		6.6/10,000	14,806					
																Females	48		6.0/10,000	27,307					
															Follow-up time among non-familial contacts	1 year						Survival analysis	Survival probability (SE)	0.9997 (0.0001)	
																2 years								0.9991 (0.0001)	
																3 years								0.9986 (0.002)	
															Follow-up time among familial contacts	1 year								0.9948 (0.002)	
																2 years								0.9842 (0.004)	

[13] KR, contact sharing roof and kitchen ("household") with the index case; K, contact sharing the kitchen; N1, next-door neighbor, not sharing kitchen or roof; N2, neighbor of neighbor

[14] new cases / 1,000 contacts (95% CI)

																3 years								0.9792 (0.005)	
Higher incidence of leprosy was found among familial contacts (FC), than among non-familial contacts, and risk increased with age.																									
(19)	Fischer EAJ et al, 2008	Bangladesh (District of Nilphamari)	1989-2003	CH	Individual	Retrospective cohort study to identify spatial distribution of leprosy patients detected in 1989-2003 and environmental risk factors associated with leprosy.	PB	1,500,000 approximately	NA	MX	11,060	I	0.49 / 1,000 PYR	Distance to road, di stance to river, distance to clinic, distance to town	Distance to road (linear)<br>Distance to road (squared)<br>Distance to river (linear)<br>Distance to river (squared)<br>Distance to clinic (linear)<br>Distance to clinic (squared)<br>Distance to town (linear)<br>Distance to town (squared)		NA	NA	NA	NA	NA	PR	RR	0.911 (0.894, 0.929)<br>0.995 (0.993, 0.996)<br>1.028 (0.988, 1.070)<br>0.998 (0.990, 1.005)<br>0.963 (0.939, 0.987)<br>0.997 (0.995, 0.999)<br>0.890 (0.866, 0.914)<br>0.990 (0.988, 0.993)	0.934 (0.915, 0.953)<br>0.996 (0.999, 1.002)<br>1.033 (0.992, 1.075)<br>0.998 (0.996, 1.012)<br>1.006 (0.981, 1.033)<br>1.000 (0.999, 1.004)<br>0.922 (0.895, 0.950)<br>0.993 (0.990, 0.995)
The study shows association of new leprosy cases rate with proximity to towns, especially in the first kilometers, and proximity to roads; no relationship was found with the proximity to a clinic or to rivers																									
(42)	Lana FCF et al, 2009	Brazil/Minas Gerais	2003-2006	EC	Municipality	The study aims to analyze the association between leprosy detection rates and Human Development Index (HDI).	PB	853	All ages	MX	NA	I[15]	NA	No	HDI	Low (0.694)<br>Median (0.694-0.750)<br>High (>0.750)						Contingency table	Chi square test Bonferroni correction: for total population (8df) and only high versus low HDI (4df)	21,51 (8df; p=0.008)<br>16,53 (4df; p=0.017)	
Municipalities with high IDH had lower leprosy detection rates than municipalities with low IDH, which suggest that cities with worst socio-economic conditions have less control of the endemy.																									
(43)	Imbiriba EB et al, 2009	Brazil (city of Manaus)	1998-2004	EC	Census tract	Ecological study on geographical distribution of leprosy and associated factors, based on 1,536 census tracts in the city of Manaus. The dependent variable was the smoothed average detection rate, categorized as 0, for < 4 cases/10,000, or 1, for ≥ 4 cases/10,000 cases.	PB	1,451,958 (population in 2001); 1,536 census tracts	All ages	URB	4,104	I	4.21 / 10,000	Cases in <15 year old children, life conditions index	Cases in <15 year old children<br><br>Life Conditions Index	0<br>1<br>2+<br>Fair<br>Middle<br>Middle-low<br>Low	NA	NA	NA	NA	NA	LR	OR		1.00<br>1.76* (1.31, 2.36)<br>2.44* (1.41, 4.20)<br>1.00<br>1.67* (1.14, 2.44)<br>3.05* (2.15, 4.32)<br>4.43* (3.14, 6.24)
Higher risk of leprosy was independently associated with higher occurrence of cases in <15 year old children and worse conditions of life																									
(37)	Durães SMB et al, 2010	Brazil (Duque de Caxias municipa lity)	2004-2007	CS	Individual	Cross-sectional study on contacts of 107 index cases	Contacts	1,040	All ages	URB	211 coprevalent cases	P		Degree of kinship, type of contact	Degree of kinship<br><br>Type of contact	Other (including non-relatives)<br>1st degree (parents, children, siblings)<br>Peridomiciliary<br>Domiciliary	87<br>124<br>48<br>163	14.3%<br>28.8%<br>12.2%<br>23.3%		522<br>307<br>347<br>482	85.7%<br>71.2%<br>87.8%<br>74.7%	LR	OR	1.00<br>2.42* (1.75, 3.35)<br>1.00<br>2.44* (1.69, 3.40)	1.00<br>2.05 (NA)<br>1.00<br>2.00 (NA)
Household contacts showed higher risk of leprosy, independent on kinship																									
(44)	Queiroz JW et al, 2010	Brazil (Mossoró municipality)	1995-2006	EC	Census tract	Study conducted on 170 census tracts, where 808 leprosy cases were selected out of 1,293 cases diagnosed between 1995 and 2006, and the dwellings were geocoded. Spatial linear regression	PB	213,841 (2000 Census); 170 census tracts	All ages	URB	808	I[16]	0,0 to 31.7 / 10,000 (average yearly detection rate, across 170 census tracts)	NA	Factors extracted by principal components analysis of ten socioeconomic variables; the higher the factor score, the better the socio-economic conditions	First factor, correlated with presence of basic sanitation (existence of piped water, presence of toilets in the house and trash collection)<br>Second factor, expressing the level of literacy and income (mean family	NA	NA	NA	NA	NA	SAR (spatial autoregressive lag model)	RR	β = -0.0978 (0.0331)<br><br><br>NA	

[15] Leprosy detection rates were categorized in low (<0.2/10000), median (0.2-0.99/10000), high (1-1.99/10000), very high (2-3.99/10000) and hyperendemic (>4/10000).

[16] average yearly detection rate, across 170 census tracts

						models were adjusted to assess the association between socio-economic factors and risk of leprosy .										income, years of schooling and number of toilets in the house) Third factor, expressing the level of poverty (lack of access to bank loaning and number of residents in the household)								β = -0.1027 (0.0240)	
Using spatial linear models, a significant inverse relationship was observed between the geographic distribution of leprosy and factors expressing socioecono mic conditions (the higher the factor score, the better the conditions																									
(24)	Feenstra SG et al, 2011	Bangladesh (Districts of Nilphamari and Rangpur)	2009	CC	Individual	Study on 90 cases and 199 controls (part of the COLEP project), on the association of selected socio-economic factors with leprosy	PB	289	5 to 40+ years	RUR	90	-	NA	Age, food shortage in the last year	Sex	Female	41		-	116		LR	OR	1.00	
																Male	49			83				1.67 (1.01, 2.76)	
															Age (years)	<10	6			22				1.00	1.00
																10-19	20			65				1.13 (0.40, 3.17)	1.17 (0.41, 3.32)
																20-29	21			27				2.85 (0.98, 8.30)	3.22 (1.09, 9.51)
																30-39	15			32				1.72 (0.58, 5.12)	1.84 (0.61, 5.55)
																40-49	11			35				1.15 (0.37, 3.56)	1.28 (0.38, 3.67)
																≥50	17			18				3.46 (1.13,10.61)	3.56 (1.15,11.02)
															Wealth quintile (score)	1	25			40				1.00	
																2	20			40				0.80 (0.38, 1.67)	
																3	16			40				0.64 (0.30, 1.38)	
																4	17			40				0.68 (0.32, 1.45)	
																5	12			39				0.49 (0.22, 1.12)	
																								0.85 (0.71, 1.02) [17]	
															Educational level (highest level of schooling ≥6 years)	High	49			113				1.00	
																Low	41			86				1.10 (0.67, 1.81)	
															Crowding (>3 people per sleeping room)	No	55			129				1.00	
																Yes	35			70				1.17 (0.70, 1.96)	
															Ever food shortage	No	30			76				1.00	
																Yes	60			123				1.24 (0.73, 2.09)	
															Food shortage in the last year	No	47			128				1.00	1.00
																Yes	43			71				1.65 (1.00, 2.74)	1.79 (1.06, 3.02)
In multivariate analysis, including sex, age, wealth quintiles, educat ional level, crowding, food shortage ever and food shortage in the last year, leprosy was associ ated with food shortage in the last year, but not with food shortage ever, and with the age groups of 20-29 years and 50+ years																									
(20)	Sales AM et al, 2011	Brazil (city of Rio de Janeiro)	1987-2007	CH	Individual	Study on contacts of 1,201 new leprosy cases, diagnosed in a national reference Centre, followed-up for 16.9 years on average. A multilevel logistic analysis was performed, with contacts variables placed at the 1st level in the model, and index cases variables at the 2nd level.	Contacts	6,158	All ages	NA	319 co-prevalent cases and 133 incident cases, diagnosed among the contacts	-	-	Age, sex, educational level and income level (in contact and index cases); blood relationship, type of association, years of association, BCG scar and BCG vaccine (in contacts); family size, bacillary index and disability grade (in index cases).	Among co-prevalent cases [18]:							LR	OR		
															Age (contacts)	≥15 years	236							1.00	1.00
																<15 years	83							0.65 (0.50, 0.84)	0.86 (0.62, 1.18)
															Sex (contacts)	Female	177							1.00	1.00
																Male	142							1.07 (0.85, 1.34)	1.12 (0.88, 1.43)
															Educational level (contacts)	>10 years	39			1,073				1.00	1.00
																4 to 10 years	25			693				0.93 (0.53, 1.65)	1.08 (0.61, 1.94)
																<4 years	255			4,072				1.50 (1.03, 2.19)	1.43 (0.96, 2.15)
															Income level (contacts)	>3 minimum wages	87			2,475				1.00	
																2 to 3 minimum wages	150			2,285				1.85 (1.35, 2.54)	
																<2 minimum wages	82			1,080				2.18 (1.50, 3.17)	
															Type of close association with index cases	Non-household				2,378				1.00	1.00
																Household	99			3,461				1.44 (1.11, 1.86)	1.33 (1.02, 1.73)
															Years of close association	<5 years	220			1,057				1.00	
																≥5 years	26			4,782				2.64 (1.75, 3.98)	
															Age (index cases)	≥15 years	293							1.00	

[17] OR for linear trend
[18] Considered as a cross-sectional analysis

																<15 years								0.96 (0.53, 1.72)	
															Sex (index cases)	Female								1.00	1.00
																Male								1.47 (1.07, 1.01)	1.05 (0.76, 1.45)
															Educational level (index cases)	>10 years				833				1.00	1.00
																4 to 10 years	17			1,459				2.53 (1.37, 4.64)	2.40 (1.30, 4.42)
																<4 years	20			3,597				3.31 (1.87, 5.58)	2.72 (1.54, 4.79)
															Income level (index cases)	>3 minimum wages	232			1,451				1.00	
																2 to 3 minimum wages	40			1,344				2.31 (1.44, 3.70)	
																<2 minimum wages	84			1,181				2.17 (1.34, 3.52)	
															Family size (index cases)	< 5 persons	73			2,264				1.00	
																≥ 5 persons	149			3,566				0.71 (0.53, 0.95)	
															Among incident cases:		179								
															Age (contacts)	≥15 years								1.00	1.00
																<15 years								0.85 (0.58, 1.24)	1.06 (0.66, 1.70)
															Sex (contacts)	Female								1.00	1.00
																Male								0.78 (0.55, 1.13)	0.79 (0.54, 1.17)
															Educational level (contacts)	>10 years				1,046					1.00
																4 to 10 years	27			686					0.40 (0.16, 1.01)
																<4 years	7			3,974					0.82 (0.49, 1.36)
															Income level (contacts)	>3 minimum wages	98			2,436					
																2 to 3 minimum wages	39			2,216					
																<2 minimum wages	69			1,055					
																	25								
															Type of close association with	Non-household				2,343					1.00
															index cases	Household	35			3,336					1.96 (1.29, 2.98)
															Years of close association	<5 years	98			1,039					
																≥5 years	18			4,667					
															Age (index cases)	≥15 years	115								
																<15 years								1.00	
															Sex (index cases)	Female								8.37 (1.12, 62.4)	1.00
																Male								1.00	1.22 (0.70, 1.76)
															Educational level (index cases)	>10 years				809				1.61 (1.03, 2.53)	1.00
																4 to 10 years	24			1,428					0.70 (0.37, 1.32)
															Income level (index cases)	<4 years	31			3,519					0.60 (0.34, 1.06)
																>3 minimum wages	78			1,427					
																2 to 3 minimum wages	24			1,311					
															Family size (index cases)	<2 minimum wages	33			1,154					
																< 5 persons	27			2,218					
																≥ 5 persons	46			3,479					
																	87								
The multilevel analysis in this study showed that lower educational level among both contacts and index cases was associated with leprosy among co-prevalent cases but not among incident cases. The study also showed that close association with index cases was associated with leprosy both in co-prevalent and incident cases.																									
(45)	Cury MRCO et al, 2012	Brazil (São Jose do Rio Preto city)	1998-2007	EC	Census tract	Territory-based study to analyze the association between leprosy incidence (as a categorical variable in quintiles) and socio-	PB	419,633 (population); 432 census tracts	All ages	URB	379	I	10.4/100,000 (cumulative incidence)	NA	Socio-economic factor[19] (obtained by principal component analysis of socio economic variables - the greater the factor value, the better the	442.8 - 835.6<br>835.6 - 1,195.8<br>1,195.8 - 1,686.8<br>1,686.8 - 2,799.9<br>2,799.9 - 8,790.9						Contingency table	Chi square	180.7 (p<0.001)	

[19] Socio-economic factor (SF), based on: average years of education of the breadwinner, average years of education of the female breadwinner, average income of the breadwinner, average income of the female breadwinner, percentage of illiterate people (E), percentage of illiterate women, and percentage of residences with five or more inhabitants.

						demographic categorical variables.									socio economic level) in quintiles	0.0015 - 0.0037								NA (p=NS)	
															Demographic density (inhabitants per km2) in sextiles	0.0038 - 0.0054									
																0.0055 - 0.0068									
																0.0069 - 0.0084									
																0.0085 - 0.0104									
																0.0105 - 0.0144									
High leprosy incidence was associated with lower socioeconomic levels. There is no association between leprosy incidence and demographic density.																									
(25)	Feenstra SG et al, 2013	Bangladesh (Districts of Nilphamari and Rangpur)	2009	CC	Individual	Study on 90 cases and 199 controls (part of COLEP project), on the association of social contact patterns and other factors with leprosy.	PB	289	5 to 40+ years	RUR	90	-	NA	Age, social contact score, food shortage in the last year	Sex	Female	41	26.1%		116	73.9%	LR	OR	1.00	
																Male	49	37.1%		83	62.9%			1.67 (1.01 , 2.76)	
															Age (years)	<10	6	21.4%		22	78.6%			1.00	1.00
																10-19	20	23.5%		65	76.5%			1.13 (0.40, 3.17)	1.38 (0.48, 4.00)
																20-29	21	43.8%		27	56.3%			2.85 (0.98, 8.30)	4.07 (1.33,12.47)
																30-39	15	31.9%		32	68.1%			1.72 (0.58, 5.12)	2.41 (0.77, 7.57)
																40-49	11	23.9%		35	76.1%			1.15 (0.37, 3.56)	1.50 (0.47, 4.87)
																≥50	17	48.6%		18	51.4%			3.46 (1.13,10.61)	5.17 (1.56,17.11)
															Social contact score[20] level 1									1.09 (1.01, 1.18)	1.09 (1.00, 1.19)
															Social contact score level 2									1.07 (1.03, 1.11)	1.07 (1.03, 1.11)
															Social contact score level 3									1.05 (0.97, 1.14)	
															Household size									0.93 (0.82, 1.05)	
															Wealth quintile	1	25	38.5%		40	61.5%			1.00	
																2	20	33.3%		40	66.7%			0.80 (0.38, 1.67)	
																3	16	28.6%		40	71.4%			0.64 (0.30, 1.38)	
																4	17	29.8%		40	70.2%			0.68 (0.32, 1.45)	
																5	12	23.5%		39	76.5%			0.49 (0.22, 1.12)	
															Wealth score (continuous)									0.75 (0.57, 0.97)	
															Educational level (highest level of schooling ≥6 years)	High	49	30.2%		113	69.8%			1.00	
																Low	41	32.3%		86	67.7%			1.10 (0.67, 1.81)	
															Food shortage in the last year	No	47	26.9%		128	73.1%			1.00	1.00
																Yes	43	37.7%		71	62.3%			1.65 (1.00, 2.73)	2.03 (1.17, 3.52)
In multivariate analysis, including sex, age, social contact scores, wealth quintiles, educational level and food shortage in the last year, leprosy was associated with a higher score for social contacts at home (level 1) and in the nearby neighborhood (level 2), with food shortage in the last year, and with the age groups of 20-29 years and 50+ years																									
(38)	Kumar A et al, 2013	India (States of Uttar-Pradesh and Haryana)	2009-2010	CS	Individual	Population survey carried out in Primary Health Care subcentres in two States (Uttar-Pradesh:UP and Haryana:H), identified through multistage random selection sampling.	HS	631,618 (rural) 172,918 (urban)	All ages	MX	355 new cases (276 in UP and 79 in H)	P	4.41/10,000 (6.91/10,000 in UP and 1.95/ 10,000 in H)	Age, sex	Area	Urban	153		8.85/10,000	172,765		Contingency table	Chi square	98.3 (p<0.0001)	
																Rural	202		3.20/10,000	631,416					
															Age	0- 9	20		203 276						
																10-19	64		203 591						
																20-29	48		137 172						
																30-39	66		98 795						
																40-49	58		69,648						
																50-59	38		38,457						
																60+	61		53,242						
															Sex	Male	183		411,910						
																Female	172		392,271						
In the surveyed area, the new case detection rate was significantly higher in urban areas compared to rural areas																									
(39)	Moura ML et al, 2013	Brazil (Mossoró city)	2006	CS	Individual and family	Survey among household contacts and neighboring contacts of leprosy patients in	Contacts	637	All ages	URB	15	P	2.4/100	N	Type of contact	Household	6		2.9/100	203		NA	NA	NA (p=0.555)	
																Neighbour	9		2.1/100	419					

[20] Social contact score: level 1, in the home; level 2, within the neighbourhood; level 3, outside the neighbourhood

						two neighborhoods with the highest concentration of leprosy cases in the study municipality.																			
There was no difference in leprosy detection rate among household contacts and individuals living in neighboring residences.																									
(26)	Murto C et al, 2013	Brazil (Maranhão State)	2009-2010	CC	Individual	A matched case-control study in endemic municipalities to examine if migration in the past five years is a risk factor for leprosy and to assess the social determinants associated with leprosy among past 5-year migrants. Cases were individuals >15 years old from national registry of leprosy and controls were selected from a national primary heath care program (Family Health Program).	HS	680 (340 cases and controls)	>15 years	MX	340	-	NA	Age, sex and clinic of diagnosis (matching variables), family (parent/child/sibling) and household contact with leprosy patient	Past 5 years migration	Yes	80			55		LR	OR	1.59*(1.17, 2.38)	1.51*(1.0, 2.28)
																No	260			285				1.00	
															Among migrants in the past 5 years:										
															Age	15–29	35			28					
																30–44	21			14					
																45–59	15			9					
																60 or more	9			4					
																Male	40			35					
																Female	40			20					
															Education	Literate	54			45					
																Illiterate	26			10					
															Type of migration	In-state (Maranhao)	45			25					
																From outside Maranhao	35			30					
															Number of times the individual migrated in the past 5 years	1	61			47					
																2	19			8					
															Zone of migration in the past 5 years	Urban	47			38					
																Rural	26			13					
															Migration for work in past 5-yrs	Rural/urban	6			3					
																Yes	46			30					
															Social network prior to migration	No	34			25					
																Always	63			39					
																Sometimes	5			1					
															Who lived with during migration	Never	11			15					
																Family	64			41					
																Co-workers	14			12					
															Mean people who lived with during migration	Other	1			2					
																	8.61			6.7					
															Mean years of migration										
															Alcohol consumption	Never drank	6.25			4.8					
																drinks currently	15			14				1.00	
																drank in the past 5 years	15			28				0.50 (0.17, 1.45)	
																use to drink more than 5 years ago	43			9				4.46*(1.43, 14.15)	
															Familial and non-familial contact	No leprosy contact	7			4				1.63 (0.32, 9.25)	
																parent/sibling/child with leprosy	33			43				1.00	
																others with leprosy (regardless of consanguinity)	24			4				7.82*(2.32, 33.38)	
																								4.99*(1.70, 16.51)	
															Household contact with leprosy in the past 5 years	Yes	23			6					
																No	20			3				5.54*(1.49, 30.46)	
															Income	≤ R$ 510	59			49				1.00	
																≥ R$ 510	38			17				2.12*(0.97, 4.71)	
															Public waste service	Yes	39			37				1.00	
																No	58			49				1.00	
															Family illiteracy	Yes	22			6				3.10*(1.10, 10.02)	

																No	32<br>39			12<br>39				2.67*(1.13, 6.51)<br>1.00	
Leprosy was associated with migration in the past five years. Among migrants, having a leprosy contact, lower income, poor public waste services, alcohol consumption and illiteracy in the family were associated with leprosy. However, education, zone of residence and lifestyle stressors were not associated with leprosy.																									
(46)	Barreto JC el at, 2014	Brazil (Castanhal municipality)	2004-2010	EC	Census tract	Spatial analysis techniques were used to determine the spatio-temporal pattern of leprosy cases in eleven districts from a hyperendemic municipality in the Brazilian Amazon region.	PB	114 census tracts	All ages	URB	499	I	25.1-97.0 / 100,000		Mean number of people per household (household density)		5.0	2.6		3.8	3.2	Mann-Withney test	U	NA (p<0.001)	
A positive correlation was found between household density and leprosy incidence. Spatial clusters of high and low detection rates were also found, as well as a spatial autocorrelation of individual cases at fine spatio-temporal scales.																									
(47)	Cabral-Miranda W et al, 2014	Brazil (State of Bahia), the all of 417 municipali ties)	2005-2011	EC	Municipality	Study of new cases of leprosy that occurred between 2005 and 2011in the all of 417 municipalities in the State, in children under 15 years old. A hierarchical conceptual model in three levels was used.	PB	417	<15 years	MX	1,674	I	0.88/10,000 (2005),<br>0.52/10,000 (2011)	'Caatinga', % of water bodies, Gini Index, average No. of dwellers p/ residence, % of urban population, % of residents born in Bahia	OLS regression<br>'Caatinga' (No vs Yes)<br>Percent of water bodies<br>Gini Index<br>Average No. of dwellers /residence<br>Percent of urban population<br>Percent of resident born in Bahia<br><br>Spatial regression<br>'Caatinga' (No vs Yes)<br>Percent of water bodies<br>Gini Index<br>Average No. of dwellers /residence<br>Percent of urban population<br>Percent of resident born in Bahia<br><br>Spatial components in spatial regression<br>Lag of 'caatinga' (No vs Yes)<br>Lag of % of water bodies<br>Lag of Gini Index<br>Lag of average No. of dwellers / residence<br>Lag of % of urban populatio<br>Lag of % of resident born in Bahia		NA	NA	NA	NA	NA	LR (OLS regression and spatial regression)	RR		-0.00 (0.41) [21]<br>0.04 (0.02) [21]<br>3.84 (0.00) [21]<br>0.43 (0.04) [21]<br>0.02 (<0.00) [21]<br>-0.04 (0.00) [21]<br><br>-1.11e-03 (0.32) [22]<br>3.85e-02 (0.05) [22]<br>3.36e+00 (0.00) [22]<br>1.22e-01 (0.63) [22]<br>1.71e-02 (0.00) [22]<br>-2.28e-05 (0.99) [22]<br><br>1.29e-03 (0.41) [23]<br>-2.24e-02 (0.52) [23]<br>1.47e+00 (0.40) [23]<br>8.63e-01 (0.04) [23]<br>3.13e-03 (0.51) [23]<br>5.94e-02 (0.00) [23]
In the regression model, after allowing for spatial dependence, relative risk of leprosy in children less than 15 years old was associated with higher percentage of area occupied by water bodies, greater Gini index and higher percentage of urban population; additional explanation was given, considering the spatial components, by a larger average number of dwellers by permanent residence and a lower percentage of residents born in Bahia.																									

[21] OLS (ordinary least squares) regression estimate (Pr(|z|))
[22] Spatial regression estimate (Pr(|z|))
[23] Spatial component estimate (Pr(|z|))

(48)	Freitas LRS et al, 2014	Brazil	2009-2011	EC	Municipality	Study on the association between demographic and socio-economic characteristics of all of the 5,565 municipalities and the average smoothed incidence rate / 100,000 of leprosy, using a hierarchical log-linear negative binomial regression analysis.	PB	190,755,799 (Brazilian population according to 2010 Census); 5,565 municipalities	All ages	MX	NA	I	9.1 (3.3, 22.8)/100,000 [24]	Region, size of the municipalities, illiteracy rate (%), urbanization rate (%), Gini index, households with inadequate sanitation (%), average of dwellers/ room, contacts investigated (%), coverage of Family Health Program (FHP) (%), cases with grade 2 disability (%)	Size of the municipalities [25]	Small 1		8.4 (3.0, 21.2)			NB	RR	1.00	1.00
																Small 2		11.7 (4.3, 27.7)					1.21 (1.13, 1.30)	1.19 (1.11, 1.29)
																Medium-sized		10.0 (4.1, 31.0)					1.29 (1.15, 1.45)	1.41 (1.24, 1.60)
																Large		8.1 (3.4, 23.8)					1.05 (0.93, 1.19)	1.67 (1.44, 1.95)
																Metropolis		11.4 (4.4, 26.0)					1.07 (0.66, 1.74)	1.92 (1.15, 3.18)
															Illiteracy rate (%)	<8		3.8 (1.5, 8.3)					1.00	1.00
																≥8 to <13		8.8 (3.5, 21.2)					2.54 (2.35, 2.74)	1.51 (1.37, 1.66)
																≥13 to <24		17.6 (7.0, 44.3)					3.76 (3.49, 4.06)	2.41 (2.12, 2.74)
																≥24		12.1 (5.2, 24.3)					2.38 (2.20, 2.57)	2.15 (1.83, 2.53)
															Urbanization rate (%)	<47		8.4 (2.6, 21.0)					1.00	1.00
																≥47 to <65		10.6 (3.7, 26.6)					1.30 (1.21, 1.41)	1.27 (1.17, 1.37)
																≥65		8.7 (3.5, 22.2)					2.21 (1.13, 1.29)	2.53 (1.40, 1.67)
															Gini index	0.50		5.8 (2.3, 14.1)					1.00	1.00
																≥0.50 to <0.55		10.2 (3.9, 25.1)					1.51 (1.42, 1.61)	1.10 (1.02, 1.18)
																≥0.55		17.6 (7.1, 40.0)					2.17 (2.03, 2.32)	1.26 (1.16, 1.37)
															Households with inadequate sanitation (%)	<6		4.3 (2.0, 10.1)					1.00	1.00
																≥6 to <16		8.6 (3.2, 20.9)					2.06 (1.91, 2.23)	1.42 (1.30, 1.56)
																≥16		13.5 (5.4, 31.3)					2.64 (2.46, 2.82)	1.34 (1.47, 1.81)
															Average number of dwellers / room	<0.51		4.9 (1.4, 12.4)					1.00	1.00
																≥0.51 to <0.57		7.1 (2.9, 16.9)					1.42 (1.31, 1.53)	1.14 (1.05, 1.24)
																≥0.57 to <0.65		10.6 (4.4, 25.1)					2.03 (1.88, 2.19)	1.25 (1.14, 1.37)
																≥0.65		17.6 (7.5, 39.7)					2.49 (2.30, 2.68)	1.41 (1.26, 1.58)
															Coverage of FHP (%)	<50		5.1 (2.4, 14.5)					1.00	1.00
																≥50 to <80		11.0 (4.3, 24.2)					1.58 (1.44, 1.74)	1.19 (1.07, 1.32)
																≥80		9.9 (3.6, 24.2)					1.43 (1.32, 1.54)	1.29 (1.17, 1.41)
															Sex ratio (male to females)	<1.0		7.7 (3.0, 17.6)						
																≥1.0 to <1.1		9.1 (3.3, 23.0)						
																≥1.1		23.9 (7.5. 64.7)						
															Average number of dwellers in permanent private households	<3.1		6.1 (1.7, 15.7)						
																≥3.1 to <3.3		9.8 (3.8, 24.2)						
															Poverty rate (%)	≥3.3		10.9 (3.9, 25.8)						
																<25		4.4 (1.7, 9.8)						
																≥25 to <43		7.7 (3.0, 21.5)						
																≥43 to <66		14.4 (4.8, 37.0)						
															Percentage of the population living in extreme poverty	≥66		13.6 (6.4, 27.8)						
																<3		4.6 (2.0, 9.7)						
																≥3 to <8		8.2 (2.9, 23.2)						
																≥8 to <20		14.3 (4.7, 35.2)						
															Average monthly household income per capita	≥20		14.6 (6.5, 30.2)						
																<R$ 260		12.7 (6.0, 25.3)						
																≥R$ 260 to <R$ 421		14.1 (4.9, 34.7)						
																≥R$ 421 to <R$ 571		8.0 (3.1, 21.6)						
															Unemployment rate (%)	≥R$ 571		4.5 (1.7, 11.1)						
																<4		4.9 (1.4, 12.7)						
																≥4 to <6		9.8 (3.9, 24.0)						
																≥6 to <8		10.6 (4.2, 28.3)						

[24] median (Q1, Q3) of SIR (smoothed incidence rate) / 100,000 inhabitants in Brazil in 2009-2011

[25] small 1 (municipality with up to 20,000 inhabitants), small 2 (municipality with 20,001 to 50,000 inhabitants), medium-sized (municipality with 50,001 to100,000 inhabitants), large (municipality with 100,001 to 900,000 inhabitants), metropolis (municipality with > 900,000 inhabitants).

															(20–20) income ratio	≥8			12.9 (5.4, 28.6)						
																<12			5.2 (2.1, 11.5)						
																≥12 to <17			7.0 (2.8, 17.6)						
																≥17 to <27			11.0 (3.9, 27.2)						
																≥27			17.0 (7.7, 39.5)						
															Percentage of coverage of the Family Allowance Programme (PBF)	<16			4.1 (1.6, 9.5)						
																≥16 to <30			8.2 (3.2, 22.8)						
																≥30			14.2 (5.8, 31.4)						
Significantly higher adjusted RRs were identified for municipalities with higher illiteracy rates, more urbanized municipalities, large municipalities compared to small ones, municipalities with higher social inequality as per the Gini index, with high percentage of households with inadequate sanitation, with greater average number of dwellers per room and with higher FHP coverage																									
(49)	Nery J et al, 2014	Brazil	2004-2011	EC	Municipality	Ecological mixed design study conducted on 1,358 municipalities, all belonging to high risk clusters for leprosy detection, on the impact of the Brazilian cash transfer program Bolsa Família (BFP) and the primary health care program (FHP) on leprosy new case detection rate. Negative binomial regressions on panel data were performed.	PB	1,358	All ages	MX	200,966	I	74.8 /100,000 (2004) to 45.6 /100,000 (2011)	Bolsa Familia Progr am (BFP) coverage (a), Family Health Program (FHP) cove rage (a), illiteracy rate (b), Gini Index (b), unemployement rate (b), urbanization rate (b), average No. of residents/ househ old (b), % of < 15 years old people (b), % of poor people (b); variable categorization at tertiles (a) or at median value (b)	BFP coverage	Low (0.0–27.75%)	NA	NA	NA	NA	NA	NB	RR	1.00	1.00 [26]
																Intermediate (27.76–48.10%)								0.86 (0.84, 0.88)	0.89 (0.86, 0.91)
																High (≥48.11%)								0.83 (0.80, 0.87)	0.85 (0.81, 0.88)
																Consolidated (>48.11% + target population coverage 100% for at least 4 years)								0.73 (0.69, 0.77)	0.79 (0.74, 0.83)
															FHP coverage	1st tertile (0, 72.02%)								1.00	1.00 [26]
																2nd tertile (72.03, 95.06%)								0.99 (0.96, 1.02)	1.05 (1.02, 1.09)
																3rd tertile (> 95.06%)								1.04 (0.99, 1.08)	1.12 (1.08, 1.17)
															Illiteracy % ≥ 20.42										1.12 (1.07, 1.18) [26]
															Gini Index ≥ 0.54										1.07 (1.04, 1.11) [26]
															Unemployement % ≥ 7.47										1.20 (1.16, 1.23) [26]
															Urbanization % ≥ 59.8										0.99 (0.93, 1.06) [26]
															Average No. of residents / household ≥ 3.6										1.04 (1.01, 1.08) [26]
															% of < 15 years old people ≥ 31.1										1.12 (1.08, 1.15) [26]
															% of poor people ≥ 27.42										1.13 (1.08, 1.18) [26]
															Only for BFP:										
															BFP coverage	Low (0.0, 27.75%)									1.00 [27]
																Intermediate (27.76, 48.10%)									0.90 (0.87–0.92) [27]
																High (≥48.11%)									0.87 (0.83, 0.90) [27]
																Consolidated (>48.11% + target population coverage 100% for at least 4 years)									0.81 (0.77, 0.85) [27]
															Illiteracy % ≥ 20.42										1.12 (1.07, 1.18) [27]
															Gini Index ≥ 0.54										1.07 (1.03, 1.11) [27]
															Unemployement % ≥ 7.47										1.19 (1.16, 1.23) [27]
															Urbanization % ≥ 59.8										0.99 (0.93, 1.06) [27]
															Average No. of residents / household ≥ 3.6										1.04 (1.01, 1.07) [27]
															% of < 15 years old people ≥ 31.1										1.11 (1.07, 1.14) [27]
															% of poor people ≥ 27.42										1.13 (1.09, 1.18) [27]
															Only for FHP:										
															FHP coverage	1st tertile (0, 72.02%)									1.00 [28]

[26] model with BFP coverage, FHP coverage and the other municipality variables
[27] model with BFP coverage and the other municipality variables
[28] model with FHP coverage and the other municipality variables

															Illiteracy % ≥ 20.42<br>Gini Index ≥ 0.54<br>Unemployement % ≥ 7.47<br>Urbanization % ≥ 59.8<br>Average No. of residents / household ≥ 3.6<br>% of < 15 years old people ≥ 31.1<br>% of poor people ≥ 27.4	2nd tertile (72.03, 95.06%)<br>3rd tertile (> 95.06%)									1.02 (0.99, 1.05) [28]<br>1.09 (1.05, 1.13) [28]<br>1.14 (1.08 ,1.20) [28]<br>1.07 (1.03, 1.11) [28]<br>1.20 (1.16 ,1.23) [28]<br>1.01 (0.94, 1.07) [28]<br>1.05 (1.02, 1.09) [28]<br>1.13 (1.10, 1.17) [28]<br>1.14 (1.09, 1.19) [28]
In multivariate analysis, leprosy new case detection rate was significantly lower in municipalities with consolidated BFP coverage, perhaps reflecting a reduction in leprosy incidence, and independently and significantly higher in municipalities with a better coverage of FHP. Also, higher rate of leprosy was significantly associated with greater percentage of illiteracy and unemployment, higher number of residents per household, larger percentage of young people and larger percentage of poors.																									
(50)	Duarte-Cunha M et al, 2015	Brazil (Duque de Caxias municipality)	1998-2006	EC	Neighbourhood	Ecological study, using neighborhoods as units of analysis, on the association of new case detection rate with selected socio- economic and health services variables.	PB	855,042 (2010 population); 40 neighbourhoods	All ages	NA	2,572	I	3.70 /10,000	Proportion of households with running water; number of reference health care units with assistance by the Leprosy Program; ratio of cases with indeterminate form / sum of cases with tuberculoid, dimorphous and Virchowian forms; ratio of multibacillary cases / paucibacillary cases; number of local case-tracking campaigns performed.	Proportion of households with running water<br>Proportion of households whose head is unemployed<br>Proportion of households with 7+ residents<br>Proportion of households whose head earns up to 1 minimum wage<br>Proportion of households with running water in at least one room<br>Proportion of households connected to general sewage network<br>Log (population density)<br>Proportion of households disposing of waste in vacant lot<br>Proportion of households whose head is illiterate<br>Proportion of households without bathroom<br>Number of local case-tracking campaigns performed<br>No. of basic health care units with assistance by the Leprosy Program<br>No. of reference health care units with assistance by the Leprosy Program							PR (log-normal model)	RR (expressed as β coefficient (95% CI))	-2.011 (-3.514 ,-0.532)<br>1.155 (-0.187, 2.491)<br>0.789 (-0.252, 1.809)<br>-0.341 (-1.145, 0.472)<br>1.223 (-3.331, 5.823)<br>0.736 (-2.273, 3.752)<br>-0.028 (-0.183, 0.131)<br>8.299 (-49.391, 65.924)<br>0.475 (-4.907, 5.669)<br>-0.015 (-12.872, 12.618)<br>0.043 (-0.020, 0.107)<br>0.014 (-0.419, 0.451)<br>0.419 (-0.013, 0.857)	-2.334 (-3.831, -0.851)<br>0.019 (-0.042, 0.081)<br>0.524 (0.092, 0.963)
Multivariate analysis shows a significant association of a higher proportion of households with piped water and a larger number of reference health care units with, respectively, a reduction and an increase of leprosy detection rate. No relevant modifications of the regression coefficients were found after allowing for spatial dependence.																									
(51)	Nobre ML et al, 2015	Brazil (State of Rio Grande do Norte)	2001-2013	EC	Municipality	Study to assess the risk of leprosy associated with socio-economic factors and the presence in the municipality of	PB	167	All ages	MX	3,927	I	Approximate average yearly incidence 9 / 100,000	Presence of a train station in the municipality (Y/N); illiter cy rate (a), per	Presence of railway station<br>Illiteracy rate<br>Per capita household income	N<br>Y	NA	NA	NA	NA	NA	LR	OR	1.00<br>6.00 (2.55,13.98)<br>1.04 (0.97, 1.11)<br>0.99 (0.99, 1.00)	1.00<br>7.92 (2.93,21.41)<br>1.10 (0.98, 1.24)<br>0.99 (0.98, 1.01)

						a railway station, a marker of old railways, active in the early 20th century. The dependent variable was the average yearly new case detection rate / 100,000 between 2001 to 2013, categorized in ≤10/100,000 and >10/ 100,000.								capita household income (a), % of people living on ≤ 70 US$/ month per capita (a), unemployment rate (a), % of houses without sanitary facilities (2000), % of houses without trash collection (2000), infant mortality (2001 to 2013) (b), vaccination coverage (2001 to 2013) (b), malnutrition of children < 1 year old (2001 to 2013) (b); arithmetic means calculated from 2000 and 2010 national censuses data (a), or from yearly measurements (b)	% of people with income										
															≤US$ 70/month									0.99 (0.99, 1.00)	0.94 (0.86, 1.03)
															Unemployment rate										
															% of houses without sanitary									1.05 (0.95, 1.15)	1.03 (0.93, 1.14)
															facilities									0.99 (0.94, 1.02)	0.99 (0.95, 1.02)
															% of houses without trash										
															collection									0.97 (0.94, 1.02)	0.97 (0.92, 1.02)
															Infant Mortality per 1,000										
															% of Vaccination coverage									1.04 (0.96, 1.13)	1.04 (0.96, 1.13)
															% of malnourished children <									1.02 (0.97, 1.09)	1.02 (0.95, 1.09)
															1year old									0.98 (0.67, 1.43)	0.95 (0.62, 1.48)
At municipality level, no association of high leprosy rate with socio-economic or public health indicators was found. However, a strong association was found with the presence in the municipality of a railway station: the intense migratory drought-related movements that occurred in the past between this state and leprosy endemic areas may explain this finding.																									
(27)	Wagenaar I et al, 2015	Bangladesh (Nilphamari and Rangpur districts)	2013	CC	Individual	Study in an endemic area aimed to identify differences in dietary intake between recently diagnosed leprosy patients and controls. Cases were selected from Rural Health Program database and controls from a random cluster sample of the population. Cases and controls were balanced for age, sex, and only one individual per household was enrolled. Social, demographic, biological and food consumption data were assessed using questionnaires, measures and a 24h food consumption recall. Analyses were conducted following a framework constituted of four	PB	152	18-50 years	RUR	52	P	NA	Age (continuous) and sex in univariate and all multivariate analyses;<br>in multivariate analyses by block:<br>Block 1:.age, sex, religion and household size;<br>Block 2: age, sex, food expenditure and self-classification;<br>Block 3: age, sex, disease other than leprosy, BCG vaccination, Body Mass Index (BMI);<br>Block 4: .age, sex, Dietary Diversity	Age (continuous) [29]	mean	35.0	(9.5)		33.3	(10.4)	LR	OR	1.02 (0.99, 1.05)	A. 1.00 (0.97, 1.05)<br>B. 1.02 (0.98, 1.05)
															Age (categorical)	15-29		25.0%			37.0%				
																30-44		32.7%			27.0%				
																45-60		42.3%			36.0%				
															Sex [29]	Male	29			48				1.00	1.00
																Female	23			52				0.69 (0.35, 1.37)	A. 0.45 (0.20, 1.00)<br>B. 0.52 (0.25, 1.10)
															Religion [29]	Muslim	40			88				1.00	
																Hindu	12			12				2.21 (0.90, 5.38)	1.00<br>2.23 (0.92, 5.46<br>A. 1.41 (0.52, 3.88)
															Household size [29]	mean No. of people eating in the house	4.6	(1.4)		5.2	(2.1)			0.83 (0.67, 1.02)	0.82 (0.66, 1.02)<br>A. 0.76 (0.55, 1.04)<br>B. 0.68 (0.52, 0.89)
															Income (BDT [30])	Household mean	5,115	(3,621)		8,177	(6,398)				
																Per capita mean	1,180	(886)		1,766	(2,011)				
															Income (log) [31]	Per capita mean	2.96	(0.27)		3.12	(0.30)			0.10* (0.03, 0.44)	
															Income variation (BDT)	mean	3,827	(2,852)		5,234	(5,719)				
															Food expenditure (BDT)	Household mean	4,545	(2,323)		6,540	(3,435)				
																Per capita mean	1,046	(530)		1,340	(841)				

[29] included in the 1st block (demographic factors)
[30] BDT (Bangladesh Thaka), 100 BDT ≅ US$ 1.28 (2013)
[31] included in the 2nd block (socioeconomic factors)

						blocks: 1.demographic factors, 2.socioeconomic factors, 3.health factors, and 4.diet-related factors.								Score (DDS) and household food stocks;<br><br>A. multivariate analysis including all significant variables in analyses by block: age, sex, religion, household size, food expenditure, occupation, BMI, DDS and household food stocks;<br><br>B. multivariate analysis including the significant variables in analysis A: age, sex, household size and food expenditure.	Food expenditure (log) [31]	Per capita mean	2.98	(0.17)		3.08	(0.18)			0.02* (0.00, 0.22)	0.03* (0.00, 0.36)<br>A. 0.02 (0.00, 0.45)<br>B. 0.005 (0.00, 0.08)
															Land ownership [31]	Landless	41			58				1.00	
																Land leaser	3			8				0.49 (0.12, 1.99)	
																Landowner	8			34				0.34* (0.14, 0.81)	
															Land size	mean size (m^2)	387	(1,214)		3,161	(6,820)				
															Self-classification [31]	Very poor	17			14				1.00	
																Poor	21			29				0.61* (0.24, 1.50)	
																Low/middle	11			35				0.26* (0.10, 0.69)	
																Middle	3			22				0.11* (0.03, 0.47)	
																Rich	0			0				-	
															Occupation of the income generator [31]	Laborer	26			28				1.00	1.00
																Shopkeeper	10			13				0.84* (0.31, 2.27)<br>0.32* (0.12, 0.86)	1.28 (0.44, 3.80)<br>A. 2.08 (0.62, 6.98)
																Other	8			25					0.44 (0.16, 1.22)<br>A. 0.59 (0.20, 1.72)
																Farmer	5			19				0.28* (0.09, 0.86)	0.24 (0.07, 0.83)<br>A. 0.47 (0.12, 1.89)
																Business	3			15				0.19* (0.05, 0.76)	0.31 (0.07,1.34)<br>A. 0.66 (0.13, 3.25)
															HFIAS[32] (score 0-27) [33]		10.2	(7.4)		6.4	(7.0)			1.08* (1.03, 1.13)	
															DDS (score 0-9) [33]	No	3.2	(1.1)		3.8	(1.4)			0.67* (0.50, 0.89)	0.71* (0.52, 0.96)<br>A. 0.83 (0.58, 1.18)
															Recent food shortage (past year) [33]	Yes	10			36				1.00	
																No	42			64				2.42* (1.07, 5.47)	
															Ever food shortage [33]	Yes	2			16				1.00	
																No	50			84				4.30* (0.93, 19.77)	
															Household food stocks [33]	Yes	25			26				1.00	1.00
																	27			74				0.38* (0.19, 0.78)	0.45* (0.22, 0.95)<br>A. 0.66 (0.29, 1.50)
DDS and household food stocks were the most important diet-related factors negatively associated with leprosy. Food expenditure per capita was also negatively associated with leprosy.																									
(52)	Castro SS et al, 2016	Brazil	2010	EC	State	The study analyzed the 27 Brazilian States, to estimate the incidence of leprosy and assess the correlation between overall incidence and social and demographic variables from Census.	PB	27	All ages	MX	NA	I	22.2/100,000	N	Mean residents per household							Pearson correlation	R^2/p-value	0.46*(p=0.0148)	
															Water supply									-0.69*(p<0.001)	
															Presence of bathroom in the house									-0.52*(p=0.0052)	
															Sex	Male			25.67/100000						
																Female			18.85/100000						
Leprosy incidence is positively associated with higher household density, and negatively associated with presence of water supply and of bathroom in the house.																									
(40)	Dabrera TME et al, 2016	Sri Lanka (Puttlam district)	2012	CS	Individual	Comprehensive household survey in the all of 166 households from a small village.	PB	753	All ages	NA	39	P	511/10,000 (overall); 183.24/10,000 (in <15 year-old)	N	Sex	Female	25			396		LR	OR	1.43 (0.73, 2.80)	
																Male	14			438				1.00	
															Age (dummy)	0 to 5	3			109				0.46 (0.14, 1.50)	
																6 to 15	11			185				1.12 (0.54, 2.20)	
																16 to 30	14			165				1.86 (0.94, 3.66)	
																31 to 45	6			133				0.79 (0.32, 1.93)	

[32] Household Food Insecurity Access Scale
[33] included in the 4th block (diet-related factors)

																≥46	5			122				0.71 (0.27, 1.86)	
															Education	Primary or lower	10			120				1.73 (0.74, 4.07)	
																Secondary and tertiary	13			271				1.00	
															Occupation (dummy)	Unemployed/retired/housewifes	13			218				1.43 (0.55, 3.66)	
																Laborers (factory/ agricultural/construction)	5			61				1.77 (0.62, 5.06)	
																Merchant/office workers	2			56				0.65 (0.14, 2.90)	
																Others	0			51				1.06 (1.03, 1.08)	
															Number of occupants in household (dummy)	1-4 members	7			167				0.71 (0.31, 1.65)	
																5-8 members	31			487				1.80 (0.81, 3.92)	
																9-12 members	1			60				0.28 (0.03, 2.12)	
															Household contact	household member with leprosy	18			81				6.69*(3.42, 13.09)	
																household member without leprosy	18			633				1.00	
The study showed that pockets of high endemicity remain present at the subnational level despite national rates below the elimination target. The study showed that having a person with leprosy in the household increased the chance of having leprosy.																									

§ CC case-control, CS cross-sectional, CH cohort, EC ecological
§§ individual, family/domicile, administrative/geographic unit, spatial/temporal cluster
§§§ PB (population based), HS (Health structure based), contacts of leprosy patients, other (specify)
† RUR (mainly rural), URB (mainly urban), MX (mixed)
†† I (incidence, new cases detection rate), P (prevalence)
††† control group, cohort baseline - cases, general population
# LN (linear regression), LR (logistic regression), CX (Cox regression), PR (Poisson regression), NB (negative binomial regression), ANOVA (Analysis of variance), other (specify)
RR, OR, PR, HR, other (specify)
*statistically significant at $\alpha \leq 0.05$
‡ calculated by the review’s Authors
PYR person-years at risk
NA (not, or unclearly, described)
NS non significant
